# Such a long journey: What health seeking pathways of patients with drug resistant tuberculosis in Mumbai tell us

**DOI:** 10.1371/journal.pone.0209924

**Published:** 2019-01-17

**Authors:** Aruna Bhattacharya Chakravarty, Sheela Rangan, Yatin Dholakia, Sonu Rai, Swaran Kamble, Tejaswi Raste, Sanchi Shah, Shimoni Shah, Nerges Mistry

**Affiliations:** The Foundation for Medical Research, Worli, Mumbai, India; Jamia Hamdard, INDIA

## Abstract

**Introduction:**

The Indian Tuberculosis (TB) Programme currently faces the dual challenges of tackling increasing numbers of drug resistant (DR) TB cases and regulating practices of a pluralistic private sector catering to TB patients. A study of health seeking behaviour of DR-TB patients in such a situation, offers an opportunity to understand the problems patients face while interacting with health systems.

**Methodology:**

Forty-six DR-TB patients drawn from 15 high TB burden wards in Mumbai were interviewed using an open ended interview tool. Interviews were audio recorded and transcribed. Pathway schematics developed from analysis of patient records, were linked to transcripts. Open coding was used to analyse these units and themes were derived after collating the codes.

**Results and discussion:**

The paper presents themes interwoven with narratives in the discussions. These include awareness-action gap among patients, role of neighbourhood providers, responsiveness of health systems, the not-such a ‘merry go round’ that patients go/are made to go on while seeking care, costs of diagnostics and treatment, and how DR-TB is viewed as the ‘big TB’.

**Conclusion:**

The recommendations are based on a preventative ethos which is sustainable, compared to interventions with top-down approaches, which get piloted, but fail to sustain impact when scaled up.

## Introduction

Tuberculosis (TB) is a disease known to mankind for thousands of years. The disease however continues to remain a scourge for patients and their families and a challenging public health problem for scientists, doctors, policy makers, programme managers and researchers. The causative organism, *Mycobacterium tuberculosis*, aided by human behaviour, has been managing to constantly outpace researchers as they come up with newer drugs, diagnostics, technology and policies to control the disease. While there are still no clear answers to tackling the disease, it is a subject which continues to fascinate and perplex medical and social scientists equally. Our exploration into the health seeking behaviour (HSB) of drug resistant TB (DR-TB) patients in Mumbai, India, is an attempt to highlight the problems patients continue to face while seeking to get cured of TB and to suggest ways to help ease these problems.

### Burden of TB in India

India was estimated to have 2.8 million cases of TB, a quarter of the 10.4 million estimated cases globally in 2016 [1, 2]. The total notified cases from the public and private sectors in India in 2016, however, was 1,938,158 cases, indicating almost 30% cases had either missed getting notified or had remained undiagnosed [3]. Apart from these missing cases, the other threat to the Indian TB epidemic is the increasing numbers of DR-TB cases, with 147,000 cases of DR-TB diagnosed in 2016 [3], Large metropolitan cities like Mumbai, which have sizeable numbers of health care providers and facilities, and a vast network of diagnostic facilities for detecting drug resistance, have the lion’s share of the country’s DR-TB cases [4].

The Revised National TB Control Programme (RNTCP) in India is positioned within an existing medical system, which is pluralistic [5]. The programme faces a huge challenge of getting the vast private sector, the first point of contact for a majority of TB symptomatics [6, 7], to notify diagnosed TB cases and refer them to the RNTCP or manage them according to national guidelines [8, 9]. In a context, where the public sector is viewed negatively by the community due to its poor access, shortage of resources, and inefficient functioning, [10, 11] and the private sector which is more accessible and has a ubiquitous presence particularly in urban areas [12], patients move between the two sectors and different private providers in their quest for a cure [7].

This shift between sectors and providers often leads to delays in the health seeking pathways for TB. Delays in TB are complex and need an in-depth understanding of patients’ health seeking pathways and not merely their first point of contact with the health systems and the time taken to get diagnosed and start treatment.

### Health seeking behaviour through a medical anthropological lens

According to the Encyclopaedia of Medical Anthropology, people generally choose the simplest form of treatment, which usually is the cheapest, most effective treatment deemed by them [13]. Only when this treatment fails, do people seek a higher level of costlier and unconventional treatments. Health seeking is a dynamic process and may involve multiple providers at the same time. Due to this, people are expected to gather information and make informed choices about the wide range of medical services that are available to them, if they are positioned within an unorganised or a dysfunctional health system.

Health seeking behaviour (HSB) is increasingly recognised as an important tool for understanding people’s preferences and decision making with regard to health care options and timing of health seeking across various health conditions. Medical anthropological studies show that traditional healing is concerned with treating the human experience of sickness and healers typically seek to provide explanations for illness and to respond to the patient, family, and community issues surrounding illness [14–17]. Biomedicine, which focuses on curing diseases, has, however, systematically undermined the importance of illness experience. This is in part responsible for the disconnect people have with scientific explanations regarding causal factors for disease, which may result in delay in help seeking, non-adherence to diagnostic and treatment guidelines, and dissatisfaction of patients and their families with professional health care [15, 18–23].

People do not respond to illness in an *ad hoc* manner; they use past experiences, accumulated knowledge, contemporary advice and referral to find the optimum strategy for obtaining the best results within the prevailing circumstances. Once inside the health system, the patient may ‘shop around’ among the providers until s/he is satisfied with the diagnosis or care received. Thus, it is imperative that people are active, rational decision makers. It is very essential in public health discourses to understand HSB as this helps in aligning public health practice and health care and health service delivery models for better health outcomes.

In order to meaningfully understand ‘why people do what they do’, there is a need to understand not only the information sources and how they are interpreted, but also the underlying, unspoken, often not so conscious assumptions in the community sustaining the cognitive process leading to their HSB.

### Health seeking behaviour in TB

Given the chronicity of the disease, HSB in TB has always fascinated public health researchers. There are several studies which have looked at HSB of TB patients [24–33]. Most of these have employed quantitative or mixed methods to look at ‘treatment shopping’ by patients, delays at various points in the care pathways, to quantify delays in patients’ health seeking pathways, explore reasons for delay, and factors associated with delays, in different countries. Paul Farmer’s [27] paper from Haiti is perhaps one of the few papers discussing health seeking in TB from a qualitative methodological stand point, bringing out reasons for patient HSB from an *emic* perspective. Of the ten Indian HSB studies on TB reviewed in 2016 [28], only one employed qualitative methodology to look into the types of delay and reasons for delays to understand complexities in health care seeking in TB [29].

Our paper is an addition to this body of literature, and provides a deeper understanding of the long and tortuous pathways of patients with DR-TB in Mumbai. It goes beyond a mere description of patient pathways, documenting delays and the shifts patients make between various providers and the private and public sectors, and attempts to analyse the reasons why patients indulge in “treatment shopping” and what causes delays at various stages of their health seeking pathway.

## Methodology

### Study setting

The study was conducted in Mumbai, the financial capital of India and one of the most populous cities in the country. Almost 62% of the city’s 12.4 million people in 2011 are estimated to live in informal slums [34]. The city reported 45,675 drug sensitive TB cases in 2017, an increase of 18% from 38,667 in 2015. In the same period, multi-drug resistant (MDR) TB cases in Mumbai increased by 36% from 3,608 to 4,891 and extensively drug resistant (XDR) TB, the more dangerous variant, increased by 21% from 556 to 670 [4, 35].

The RNTCP has been functioning in Mumbai since 1999, after being introduced as a pilot project in one municipal ward in 1993, and extended to two more wards in 1995 [36]. It offers diagnosis and first line treatment services for drug sensitive TB. Services for the management of MDR-TB were introduced in 2007, which have expanded over time [37]. At the time of the study, TB diagnosis and treatment were made available through a huge network of public health facilities catering to the entire city through 116 Designated Microscopy Centres and 348 DOT Centres.

Cartridge Based Nucleic Acid Amplification Test (CB-NAAT or GeneXpert) for detecting resistance to Rifampicin, a key drug in TB treatment resistance, was performed at 19 designated laboratories in the public sector and three private laboratories which provided services to the RNTCP. Additionally, there were laboratories under the Initiative for Promoting Affordable and Quality TB Tests [38], which provided GeneXpert to private physicians for INR 1000 (USD 14.60) per test. Pre-treatment evaluation tests, which are mandatory before initiation of DR-TB treatment, were carried out either at secondary care hospitals in the public sector or in private laboratories. At the time of the study, no reimbursement of costs for these tests was made to patients.

The protocol followed by the RNTCP for diagnosis and treatment of DR-TB was as follows: patients suspected to have DR-TB were offered GeneXpert. Those who were found to be Rifampicin resistant were advised to undertake pre-treatment evaluation tests (blood tests, x-ray, and evaluation by a psychiatrist). Following the tests, they were referred to any of the four DR-TB centres for starting standard treatment for MDR-TB. Follow up sputum cultures were performed at regular intervals and if the culture at the sixth month was positive, the sample was sent to the National Reference Laboratory for second line drug susceptibility tests. If the reports suggested pre-XDR or XDR-TB, patients were started on XDR-TB treatment [39].

Bedaquiline was introduced in India in March 2016. Around 600 cases were to be started on treatment at six centres across the country. In Mumbai, one of the four medical college hospitals was the implementing site. Cases were evaluated through susceptibility tests for a panel of nine drugs. To receive Bedaquiline, patients had to be hospitalized for two weeks at the TB hospital and continue treatment at the Health Posts located near the patients’ residence. For any drug related adverse event, patients had to attend the DR-TB centre to which they were attached. For specialist consultations, patients had to go to a medical college hospital. Adverse drug reactions were managed at one of the medical college hospitals.

Apart from the RNTCP, TB was also managed by the vast pluralistic private health sector in Mumbai [40]. Most private practitioners trained in Western medicine diagnosed TB, and prescribed first line TB treatment, with or without guidance from consultant physicians. Drug resistant TB in the private sector was mostly diagnosed and treated by respiratory physicians and larger hospitals. Patients who could not afford the cost of diagnostic tests or second line drugs were referred to the public sector [41, 42].

At the time of the study, a Public Private Interface Agency (PPIA) intervention was being piloted in 15 high TB burden wards. The aim was to identify TB patients early, and refer them to appropriate testing and treatment centres, thereby increasing access to TB management services for patients seeking care in the private sector. A network of willing private practitioners were involved in the PPIA—2,713 formal (trained in Western medicine) and informal (trained in Indigenous systems of Medicine) private practitioners acted as ‘spokes’ and identified TB patients, and 593 specialists, practitioners with large practices and hospitals acted as ‘hubs’ and confirmed TB diagnosis and prescribed first line treatment [38]. Patients consulting PPIA practitioners were offered GeneXpert and X-rays free through a voucher system; first line drugs were also provided free through enlisted pharmacies, which were reimbursed on a monthly basis. Patients had to bear the consultation costs which ranged from INR 300 to 500 (USD 4.38 to 7.30) per visit. Once DR-TB was suspected or diagnosed, patients were referred to the DR-TB centre in the public sector. (Personal communication with Dr. Dholakia-Consulting Chest Physician at FMR).

### Study population

All the DR-TB cases were drawn from high TB burden municipal wards in Mumbai. This qualitative phase was nested within a larger study, undertaken to document TB patient pathways two years after the PPIA intervention was implemented.

In this paper we present and discuss the analysis of HSB and care pathways of 46 DR-TB cases identified during this study. The TB care pathway captures a patient’s journey from onset (or her/his recognition) of symptoms to diagnosis of TB and starting current anti-TB treatment. Reconstruction of such pathways is a corroborative process where a researcher stitches together information from different sources, which are often not completely congruent. On one hand, TB pathway studies create in-depth understanding of health seeking by elucidating the ‘whys’ and ‘how’ of HSB, while on the other hand, they fall short in terms of accuracy of pathway duration. As no one will remember the exact onset of initial symptoms as common as cough or fever or a combination of these, this poses a challenge in ascertaining the exact time of onset of symptoms and the patient’s recognition of these. This is the most crucial aspect for a medical anthropological study on health seeking pathway [43]. Hence our main aim in this analysis, did not involve measuring pathway durations or comparing patients’ pathways, but to see each pathway in its own contextual reality to arrive at the reasons for HSB.

### Data collection

A household survey was conducted by a collaborating partner (Sambodhi Research and Communications Pvt. Ltd., Noida) in 15 high TB burden municipal wards, out of a total of 24 wards in Mumbai. Pulmonary DR-TB cases who were diagnosed and were on treatment in Mumbai at the time of contact were included. Patients who met the inclusion criteria for the study were contacted over phone and asked about their willingness to participate in an in-depth interview. Those who expressed willingness were asked to suggest a convenient place and time for the interview. In case of patients below the age of 18 (minors), the parents/guardian were requested to be present along with the patient. Four researchers, who had experience in community-based research, conducted the interviews using an open ended interview schedule. The researchers were trained to conduct in-depth interviews by a team, including SR (Public Health specialist), and in TB diagnosis and treatment by YD (Chest Physician). Before starting the interview, researchers provided information to the respondents about the study and its purpose, discussed details of the interview process and obtained their consent. All interviews took place in private, and in places preferred by the interviewees. Two researchers were present for each interview (in the roles of interviewer and note taker) and all interviews were audio recorded and field notes maintained. All included patients were on DR TB treatment at the time of interview.

Forty three interviews were carried out in Hindi and three in Marathi. Once data were collected, all interviews were checked for quality by SKR (Public Health), SK (Zoology), TR (Population Science), SS2 (Bioinformatics), YD and SR. Researchers corroborated audio data with medical reports and prescriptions available with the patients (and their families), with help from YD. Data were collected between February and June 2017.

Three of the four researchers namely, SKR, SK and TR continued to participate in the analysis of the qualitative data. All members of the team had proficiency in English, Hindi and Marathi, except ABC and SKR, who were non-conversant in Marathi. All audio data were transcribed in Hindi and the three interviews conducted in Marathi, were also translated, in order to retain nuances of the local language. Transcriptions were done by SKR, SK, TR, and supervised by ABC (Medical Anthropologist). A detailed chart was created by ABC to ensure that researchers did not transcribe interviews conducted by them. Note takers from the interviews acted as co-transcribers and reviewed completed transcriptions. This was done in order to achieve objectivity and reduce bias.

A pathway schematic was drawn from analysis of all patient records: laboratory reports, doctor’s prescriptions and pharmacy bills related with the current episode of TB, and each milestone was ascertained on the basis of corroboration from patients’/family/relatives of patients narratives (interview transcripts) as well as these documents. Each transcript was linked with the pathway schematic created by SS2, in a single document. The complete unit was further analysed using qualitative research methods. Transcription, translation and collation of pathway schematic were completed between October and December 2017.

### Data analysis

Each document (transcript along with pathway schematic) was coded twice, by separate coders (ABC, SKR, SK, and TR). Open coding [44] was used for analysis since a code book would have limited incorporation of point of views emerging from coders who came from different disciplines. Broad themes (Table 1) were derived after collating all open codes using principles of grounded theory [45]. Manual coding was employed and the process of ‘coding and analysis’ was completed between January and February 2018.

**Table 1:** Analytical framework.

Open Coding	Axial Coding	Selective Coding (Broad Themes)
**TB Treatment pathway**		Relationship between Awareness and Suspicion of TB and Affirmative Action
Diagnosis	**What is Awareness for TB**	
Referrals and Health Systems		
Confusion around MDR diagnosis, paid test, referrals and lack of it	Tuberculosis in the community, Tuberculosis in the family, Tuberculosis in the past	
Side Effects		
**Health seeking behaviour**		Patients’ Health Seeking Pathway: the role of ‘Neighbourhood’ Providers ad Facilities
Symptoms and accessing health systems	**Why do they not perceive their symptoms? When do they start to suspect TB?**	
When they access health systems, picking up the symptoms	The crucial point for beginning of TB HSB	
First point, nearest provider		
shopping—going from one provider to another	**Patient not confident and Health Systems not responsive and then there is drug resistant TB**	
private sector, fees, out of pocket payment, not free	Over dependent patients and Health Systems failing to deliver	Responsiveness of Health Systems to TB Symptomatics and Delays in Diagnosis
**Delay**		
Delay in Diagnosis	**Patients could have been screened earlier if providers had taken detailed case history**	
Delay in starting Symptomatic relief, not screening for TB		
Missing obvious signs and symptoms, Missed opportunity	**‘Do patients shop for treatment or do providers let them shop?’**	Patients’ TB Care Pathway: A not so ‘Merry go round’
Referrals and Health Systems		
Delay due to poor history taking	**So many facilities and so many providers yet patients go ‘round and round’ for seeking TB treatment**	
Old case, yet accessing Health systems is a problem		Availability and Costs of Diagnostic and Treatment Facilities for TB
**Role of Health Systems**		
Symptomatic relief, not screening for TB	**Role of ‘providers and facilities in the neighbourhood’**	
Missing obvious signs and symptoms, Missed opportunity		
Referrals and Health Systems	**MDR is the ‘big TB’**	DR-TB: The ‘big-bad’ TB
Old case, yet accessing Health systems is a problem		
Confusion around MDR diagnosis, paid tests, referrals and lack of it		

All team members worked in tandem, with frequent team meetings and debriefing sessions. ABC led data analysis and wrote the first draft of the manuscript. SS (Public Health) and SS2 developed materials on the background and reviewed this work from time to time. NM (Biologist and Research Scientist) gave overall leadership to this project and along with SR and YD, provided important inputs during data analysis. All team members contributed to writing and finalising this manuscript.

### Ethical consideration

Written consent was obtained from all the patients willing to participate in the study. The consent included participation in the interview, digital audio recording and note keeping of the patients’ responses, examining and scanning all relevant TB treatment-related documents, and permission to publish anonymised data (including quotes) in any report, journal, etc. Patient anonymity was maintained by identifying each patient by a unique identification number. In case of minors, consent was obtained from their parents/guardians.

Ethical approval was obtained from the Institutional Ethics Committee (IEC) of the Foundation for Medical Research (vide IEC no. FMR/IEC/TB/01/2013).

## Results and discussion

In our analysis of the 46 DR-TB cases, we looked beyond the oft discussed topics of HSB in TB and that of a TB pathway study—of delays at various stages of health seeking, contribution of patients and providers to these delays and the numbers and types of providers consulted by patients. We registered the TB HSB characteristics and asked *why do these characteristics repeat over and over again*? For example, in the work of Paul Farmer in Haiti [27], *Robert David*, the 19-year-old boy, goes through misdiagnosis, delays in his TB HSB pathway; and then we see similar patterns in studies by Chakraborty, et al. [46], Petros, et al. [47], Mistry, et al. [32] and, McDowell and Pai [40] from Indian settings. As public health researchers, we wish to see an end to these patterns in favour of an effective and efficient health systems response in terms of screening, diagnosis and treatment, which will result in reduced transmission. Thus, this work attempts to highlight scopes for health systems in recognising the missed opportunities for early diagnosis, effective screening and treatment as well as for preventative measures for TB in the community. In the following sections, discussions based on thematic analysis are presented. Some themes are presented interwoven with narratives from interviews in order to bring home discussions which cannot be captured just with quotes (*Adopted names of patients have been used for illustrating TB pathway narratives)

### Relationship between awareness and suspicion of TB and affirmative action

TB was not an unknown disease for the patients in this study. They came to know about it from other TB patients—in their neighbourhood, workplace, and families—and even their own TB in the past, as well as from the Information, Education, Communication (IEC) and Behavioural Change Communication (BCC) efforts of the RNTCP. There, however, seemed to be a sizeable gap between people’s awareness about TB and the desired actions from them with regard to recognition of TB symptoms, suspicion of TB and prompt health seeking. This is what led patients into a complex and confusing situation of denial, fear, a sense of feeling powerless and helpless, along with stigma and vulnerability.

Raju* has lived with his family in one of the Mumbai slums since his birth. He was working as a cashier in a super market when he started to have symptoms of TB. He has seen TB from close quarters—two of his brothers have TB and are on medication. He also mentioned about not completing treatment when he was diagnosed with TB 15 years back. Yet, even though he suspected his symptoms may be because of TB, he took medicines suggested by a local chemist and waited till his symptoms got worse, before he visited a health facility.

(Fig 1)

**Fig 1:**
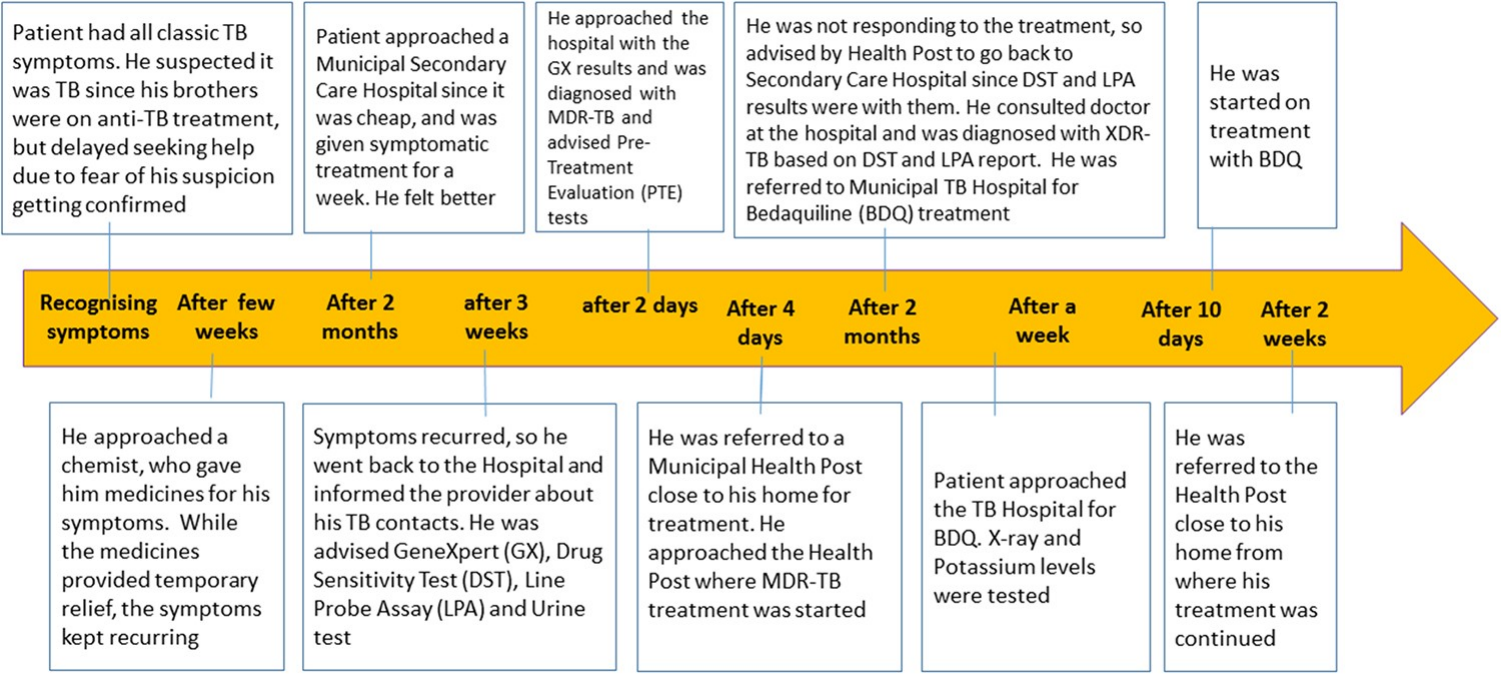
‘Raju’ Male 25 years,with past history of incomplete TB treatment and two family members with TB (Total Pathway around~ 6 months).

The stories of our patients suggested that mere awareness about the disease, did not result in quick and appropriate action from patients and their families; patients often waited for their symptoms and/or their condition to worsen. This could be because symptoms like cough, fever, fatigue, loss of appetite etc., did not trigger suspicion of TB among patients. What was surprising, however, was that even patients with a previous history of TB, did not suspect their symptoms could be due to TB. The study by Mistry et al. [32] reports very similar findings: it highlights that the mean duration of seeking care for the first time after development of symptoms was similar in new and retreatment patients. This could be because they had forgotten about their experiences with TB or because they believed they would not get TB again, since they had taken complete treatment. This could also be because the health systems had failed to provide this information to patients on completion of treatment. Patients could also be scared to admit even to themselves that what they had could be TB, for they could not visualise going through the experience of taking TB treatment again. Raju* admitted he did not want to further burden his mother, who was already struggling to look after his brothers who had TB, which is why he delayed consulting a doctor and getting tested.

Sanju* says he consulted a doctor only after he had haemoptysis, which got him worried… two to three months after he started getting his initial symptoms.

[Retreatment Case, Male, 25y, 2 months’ pathway]

Saru*, is a married woman, who lives in a slum. When she started to observe symptoms like cough, breathlessness and loss of appetite, she attributed them to going out in the rains. When she visited the first provider, she did not think it could be TB even though she had taken TB treatment 20–25 years back.

(Fig 2)

**Fig 2:**
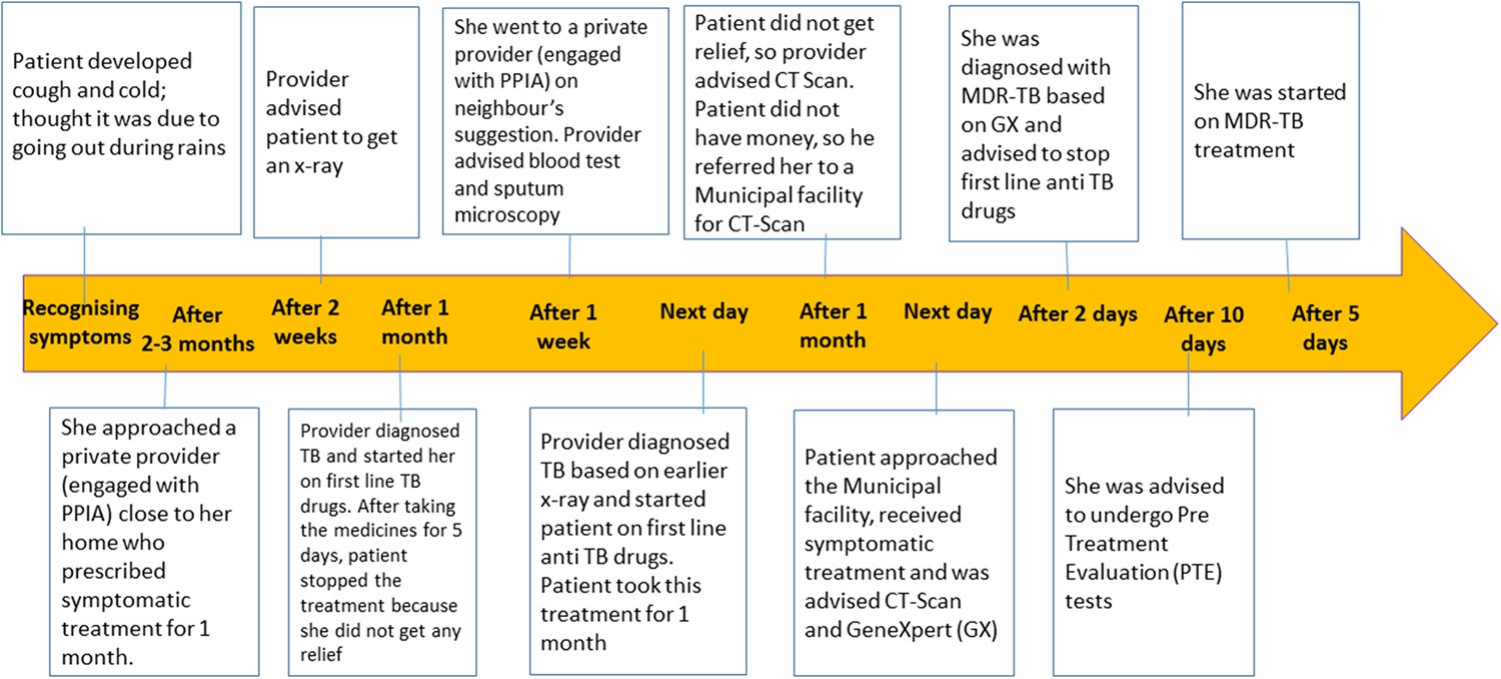
‘Saru’Female 48 years with, past history of completing Anti-TB treatment 25 years back(Total Pathway~3.5 months).

Firdoz* earned his living doing embroidery work. He had TB in 2014 and had taken medicines for six months. His daughter was also on TB medicines when he was being interviewed. Yet, he took his initial symptoms of cough and mild fever, which started to appear during the previous winter, as that of common cold.

(Fig 3)

**Fig 3:**
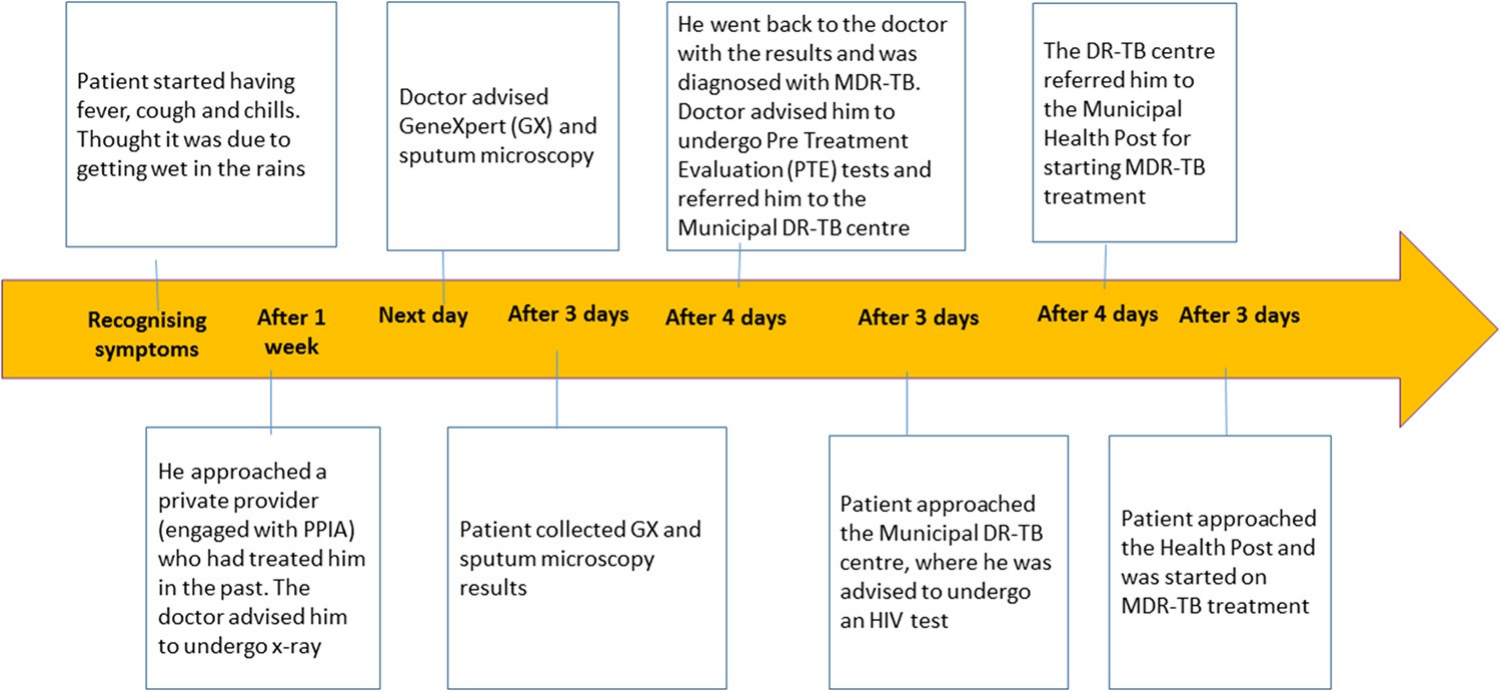
‘Firdoz’ Male 57 years with past history of completing Anti-TB treatment.

Abraham*, thought his cough was due to his smoking. When he started getting chest pain, his son got him admitted in a hospital. The doctor prescribed some medicines and sent him home after a day. He felt better with these medicines. Three to four months later, the pain recurred and it was quite severe. It was then that he suspected he was unwell. But then too, he attributed it to his being very busy looking after his hospitalised wife and neglecting himself. It was only when he found it difficult to walk due to weakness that he went to see a private doctor. By then he had lost more than 10 kg.

[Retreatment Case, M, 62y, 6 months’ pathway]

When Reshma*, a school student, started to have symptoms, TB was the last thing her family suspected. Reshma’s mother says. “…she was going to school and every evening she would get fever. He (Reshma’s father) said it must be cold; we will get her checked up. But we didn’t pay too much attention. Her exams were on, we allowed her to go to school and thought, let her exams get over.” Her parents were taken by surprise when Reshma was diagnosed with drug resistant TB; her mother remarked ‘we just could not believe it!’

[New Case, F, 15y, 2 months’ pathway]

What started with innocuous symptoms turned out to be DR-TB, which took *Reshma’s* family by surprise; they did not expect their young daughter to have something so serious, especially when she had always been healthy. Another patient, who had completed TB treatment in the past, thought she would never get TB again, but she was in for a surprise when she was diagnosed with DR-TB: ‘*I thought you get cured when you complete your treatment once*.*’* These narratives highlight failures of the health systems in disseminating clear and complete information, in a form which the community can comprehend easily, without raising fear and panic. We often argue about patients’ educational qualification to explain the knowledge-action gaps [48]. We do not, however wish to justify the socio-economic vulnerability for contracting TB: everyone is vulnerable to TB when we exist within weak health systems and congested urban environments.

### Patients’ health seeking pathway: The role of ‘neighbourhood’ providers and facilities

“First we go to a private facility; it’s here only, very close to home”

[Retreatment Case, M, 50y, 1 months’ pathway]

Tuberculosis care pathways almost always began with a neighbourhood provider. This is a matter of convenience and the normative HSB of a community. Early symptoms of TB which were not severe, were most often seen as ‘*ordinary*’. It was normal for patients with such ‘*ordinary*’ symptoms to visit the ‘*family doctor*’ in the vicinity to seek treatment. These facilities and providers represented a wide range, from an unregistered practitioner, a municipal health centre, a private practitioner practicing modern Western medicine to a practitioner of AYUSH (Indian Medical system Ayurveda, Yoga, Unani, Siddha, Homeopathy), demonstrating the existing medical pluralism in the community. These practitioners in the neighbourhood provide treatment or referral services for the community and have long been identified as an important part of the health system from the public health perspective [40]. For TB control, given the thrust on public-private partnership, both formal and informal providers and their networks play a significant role [25].

There were various reasons why people preferred going to the neighbourhood clinic: there was a sense of familiarity and convenience since these clinics were open in the evening, they were more accessible and easy to use compared to government facilities. In addition, travel costs for visiting such neighbourhood facilities were minimal.

When Asha* fell ill, her family member approached a provider near their home. The family preferred going to this facility as it was open for long hours and in case of fever and emergencies, they could access this facility quickly. “Otherwise, for the other hospital (referring to a Municipal Secondary Care Hospital) we need to go early in the morning, and once we go there—they will give two tablets and they will tell us to visit next day. And we need to take auto rickshaw to go there; the meter charge is a minimum of 80–90 rupees [USD 1.17–1.31] … one way it is 70–80 rupees [USD 1.021.17-]”.

[New Case, F, 14y, 3 months pathway]

Whereas, Majnu* offered a different explanation. “For not so serious ailments, we go to any private clinic which are here (there are many nearby). These are close by, and they give medicines as well. There is no municipal facility nearby….. If we go to S (a Municipal Hospital a little distance away) then it takes the whole day—from morning to evening. It takes a lot of time, and because of my illness (I) can’t sit there (and wait)”.

[New Case, M, 32y, 14 months’ pathway]

The community’s dependence on the ‘neighbourhood doctor’ further stresses their potential roles for TB control in the community. In a typical TB care pathway, these providers and facilities from the neighbourhood are associated with ‘delay’ and often blamed for not providing the right treatment or correct referral [49, 50], because they too fall within the hierarchy of health systems. It is this recognition that has led to the various attempts across the world to involve private providers in national TB control programmes, through Public Private Mix (PPM) and PPIA [38].

### Responsiveness of health systems to TB symptomatics and delays in diagnosis

Saru* says “we go to the clinic and they give medicines".

The problems of patients did not end with contacting the health systems. Patients (and the community at large) are positioned in a vulnerable situation juxtaposed to the hierarchical health systems [51]. This was reflected in the behaviour of our patients. They exhibited a strange mix of confusion, fear and tentativeness in approaching health care providers, and confidence in the ability of providers to treat their illness. What was surprising, however, was the similarity in the behaviour of new patients and those who had TB in the past and had prolonged interactions with TB care providers and facilities.

Saru’s* words, *‘we go to the clinic and they give medicines’* were steeped in so much confidence and faith in the system, that she did not bother to recognise her symptoms and link them to her previous episode, remember the medicines she had taken for her treatment in the past, and for that matter the doctors/facilities she had visited the previous time. The health systems, however, had their own problems too, and often fell short in meeting patients’ expectations. It took Saru* (pathway presented below) more than three months to get the right diagnosis of DR-TB and to start treatment.

Even Reshma’s* parents, who were shocked to know their daughter had contracted DR-TB, expressed their complete faith in the system. It was evident when they said “The Government has done extensive research on TB and there are medicines which cure TB; it (TB) is no longer a threat”.

[New Case, F, 15y, 2 months’ pathway]

Like Saru*, a majority of the patients interviewed had experienced delays in diagnosis. This corroborates the findings reported by a study in Mumbai: diagnostic delay (time from seeking care for the first time to receiving the diagnosis) for TB patients had contributed to 55% of the total health seeking pathway [32]. For several of our patients, providers (both government and private) had overlooked initial symptoms and treated them with drugs to provide symptomatic relief. This was true for Rekha* (pathway presented below) and a few others, whose first source of help seeking was a ‘family doctor’, who, at times, had also been responsible for treating their earlier episode of TB. Had these providers spent a little more time, taken detailed case histories, and suspected TB among their patients, who came from high TB burden localities, these delays could have been avoided. Das et al’s study in Delhi reports similar problems among private practitioners with regard to managing patients presenting with TB symptoms: they spent six minutes on an average with patients and their history taking was poor [52].

Rekha’s* care seeking pathway demonstrates how her family doctor failed to suspect TB, *even though he had treated her earlier episode of TB*. *Her total pathway duration of close to a year also involved several visits to public sector facilities before she was diagnosed with XDR-TB*.

(Fig 4)

**Fig 4:**
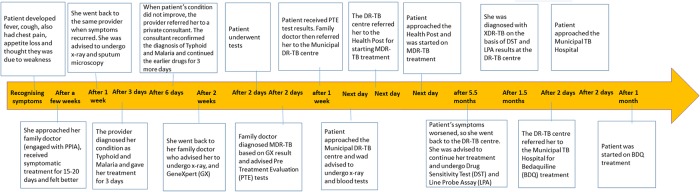
‘Rekha’ Female 24 years, with past history of completing Anti-TB treatment (Total Pathway~ 11 months).

### Patients’ TB care pathway: Not-such a ‘merry go round’

Studies point to TB care pathways involving many providers and facilities [25, 40]. Mistry, et al.’s study in Mumbai slums reported that DR TB patients had consulted as many as ten providers in their entire care pathway [32]. Our analysis of the care pathways of the 46 patients too brought us face to face with the long and tortuous journeys these patients had undertaken, punctuated by visits to several providers/health facilities and laboratories, while seeking care, what is often referred to as ‘treatment shopping’. Delays in diagnosis and treatment initiation are often attributed to such patterns of health seeking. When we looked at the reasons for these patterns and the consequent delays, we saw a different picture emerge, which led us to conclude that labelling all changes in providers as ‘shopping’ would indicate wilful patient behaviour, which was not the case.

There were a few patients who had changed providers because they did not get better in two or three days. In several cases, it was because they did not have the patience or the luxury of time to wait and get better, because they needed to go back to work, or go to their native village for attending social functions, or attend school and prepare for exams. They hoped the next doctor would give them ‘stronger’ medicines to make them better. Sometimes, patients were forced to change the provider because they could not afford the cost of the tests their providers advised, or the medicines they prescribed. At times, it was the lack of clear communication by the provider, about the need for a particular test or medicine or the need for follow up after completion of the course of prescribed treatment. In such situations, the patients had assumed that the doctor’s treatment had failed and hence had gone to another provider.

Providers often blame TB patients for being ignorant, or in denial and delaying seeking care [53, 54]. Several of our patients, however, had delays attributable to the health systems; some of these delays could not be simplified, given the maze of facilities and providers, patients needed to traverse to get diagnosed and receive treatment for DR-TB. Rekha’s* care seeking pathway (Fig 4) demonstrates both these issues well.

#### Navigating health systems

The health seeking pathway of each patient was unique and these pathways allowed us to see different contexts even while these were positioned on the same health systems. In this section we use patient pathways to elaborate on this aspect, and understand the network of health systems which surrounded these 46 DR-TB patients, and the delays they experienced, not just in terms of duration but also in spatial context. Nevertheless, instead of measuring these pathways as long and short, we need to view each step in the pathway matrix, which shows the trials they endured to get the right treatment.

Firdoz*, *the 57 year old embroiderer*, *had become too weak to work and had difficulty in walking*. *He had trained his sons to do his work*, *but they did not get enough work to run the household smoothly on their own*. *His was the shortest pathway of a month*, *because he was lucky to first consult a private provider who was part of the PPIA network*. *He was suspected early and advised the appropriate tests to diagnose DR-TB*, *and then referred to the correct public sector facility for receiving DR-TB treatment*.

(Fig 3)

It can be argued that this was the best the health systems can do for a patient with DR-TB. However even a short pathway of a month like in the case of *Firdoz*,* which involved going from one facility to another during the monsoons in Mumbai, was not easy for a sick man, who was barely able to walk. There definitely is a need for the redesign of referral systems minimizing patient movement and thus promoting patient adherence, by simplifying processes in the diagnosis and treatment of DR-TB.

This is Raju’s* care pathway (Fig 1). *In the six months it took him to be started on XDR-TB treatment*, *he had consulted six providers*. *After being diagnosed with DR-TB at the DR-TB centre*, *he was referred to a Health Post near his home for treatment initiation*. *When his condition worsened after three months of treatment*, *he was referred back to the DR-TB centre for further evaluation*, *found to have XDR-TB and referred to the TB hospital for admission*, *and evaluation for fitness for treatment with Bedaquiline*. *Once treatment was started*, *he was referred back to the Health Post for continuation of treatment*.

**Fig 5:**
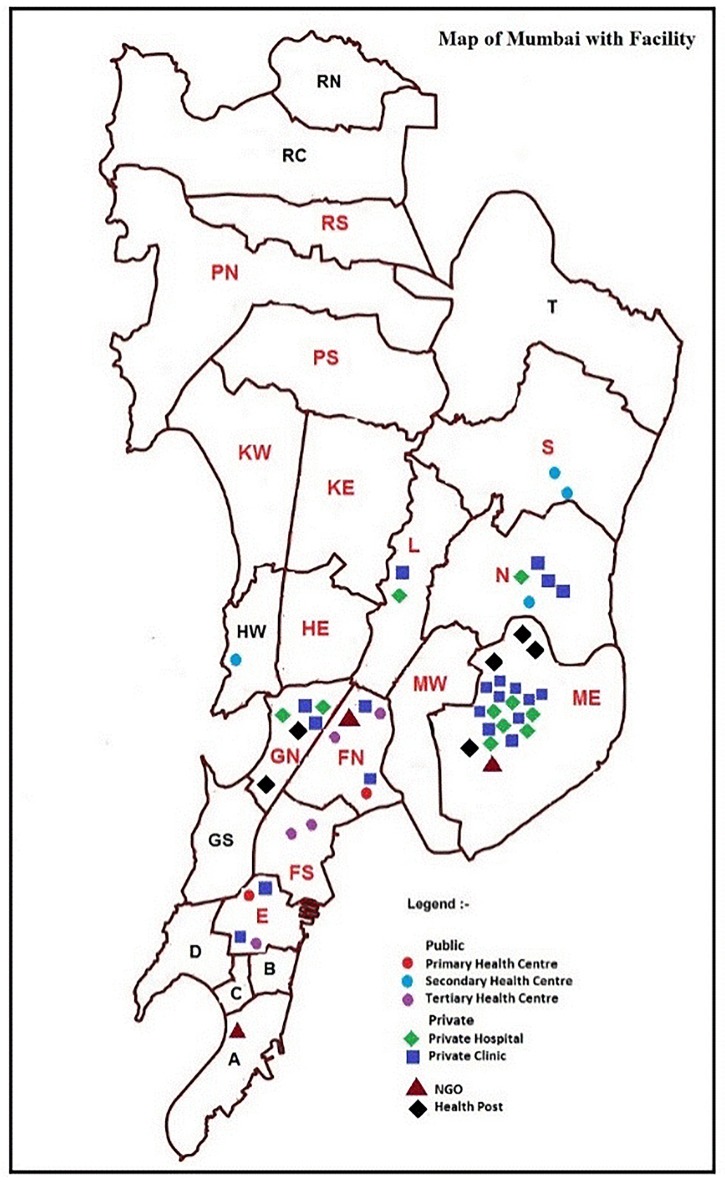
Map of Mumbai with facilities with wards highlighted.

This scenario played out in several patients, who were required to go back and forth between DR-TB centres, the TB hospital and peripheral health facilities. While Raju* was smart, had some experience with his brothers’ treatment and knew the reason he was being referred to and fro between various facilities, the same was not the case in several other patients. They were unclear about why they were made to undergo the same tests at different places and at different times, referred to different facilities and subjected to changes in their treatment regimen in their extremely weakened state. Our patients accessed several health facilities in Mumbai (Fig 5), which were meant to work like a network to support different aspects of a TB patient’s pathway, from offering diagnostics, consultations with specialists, to dispensing medicines; but patients were unaware about these links. They could only distinguish between the public and private sector facilities. They rarely understood the difference between doctors trained in Western medicine and those trained in the indigenous systems of medicine, and general practitioners and specialists, leave alone distinguish between a private provider engaged with the PPIA network and one who was not. They were not aware of primary, secondary and tertiary care facilities within the public sector; nor did they understand that only a few centres were designated to manage DR-TB, or that assessment for suitability for Bedaquiline could only be done at the TB hospital. Hence the patient’s journey remained confusing and ambiguous as s/he went from one facility to another, straddling the private and public sectors and following instructions given by health facilities and providers, which they seldom understood clearly. In the process, the patient submitted him/herself to the hierarchical system, merely looking for relief from symptoms of TB.

Bina’s* pathway demonstrates this very well. Her pathway duration of more than four months could be seen as a typical case for health seeking behaviour in TB, but the pathway schematic fails to bring up the spatial pathway across the city of Mumbai, where she sought treatment for TB from six different facilities/providers spanning about four and half months. She could not understand why she was made to run between so many facilities, all within the public sector.

(Figs 6 and 7)

**Fig 6:**
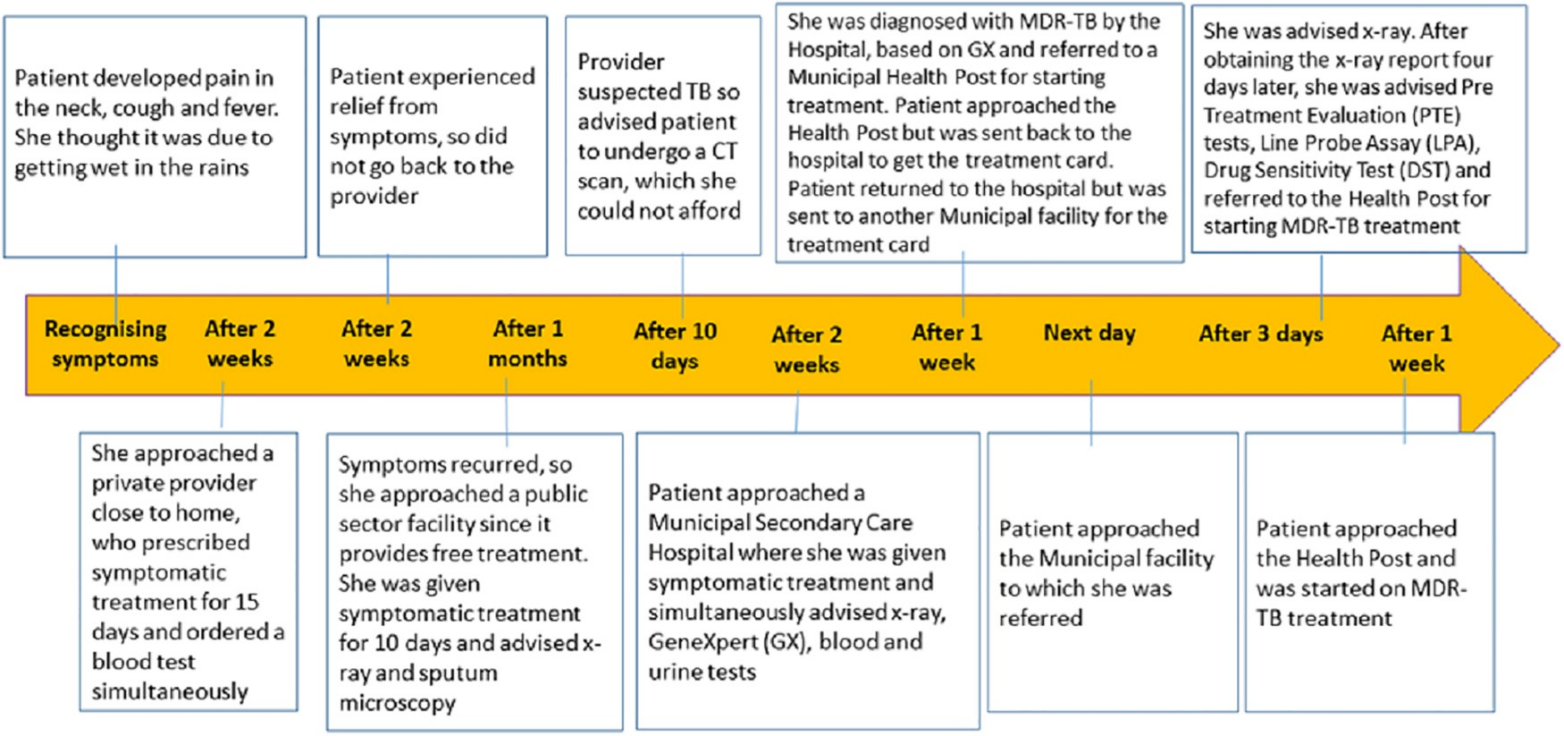
‘Bina’ Female 24 years with history of TB 8 years back (Total pathway ~4 months).

**Fig 7:**
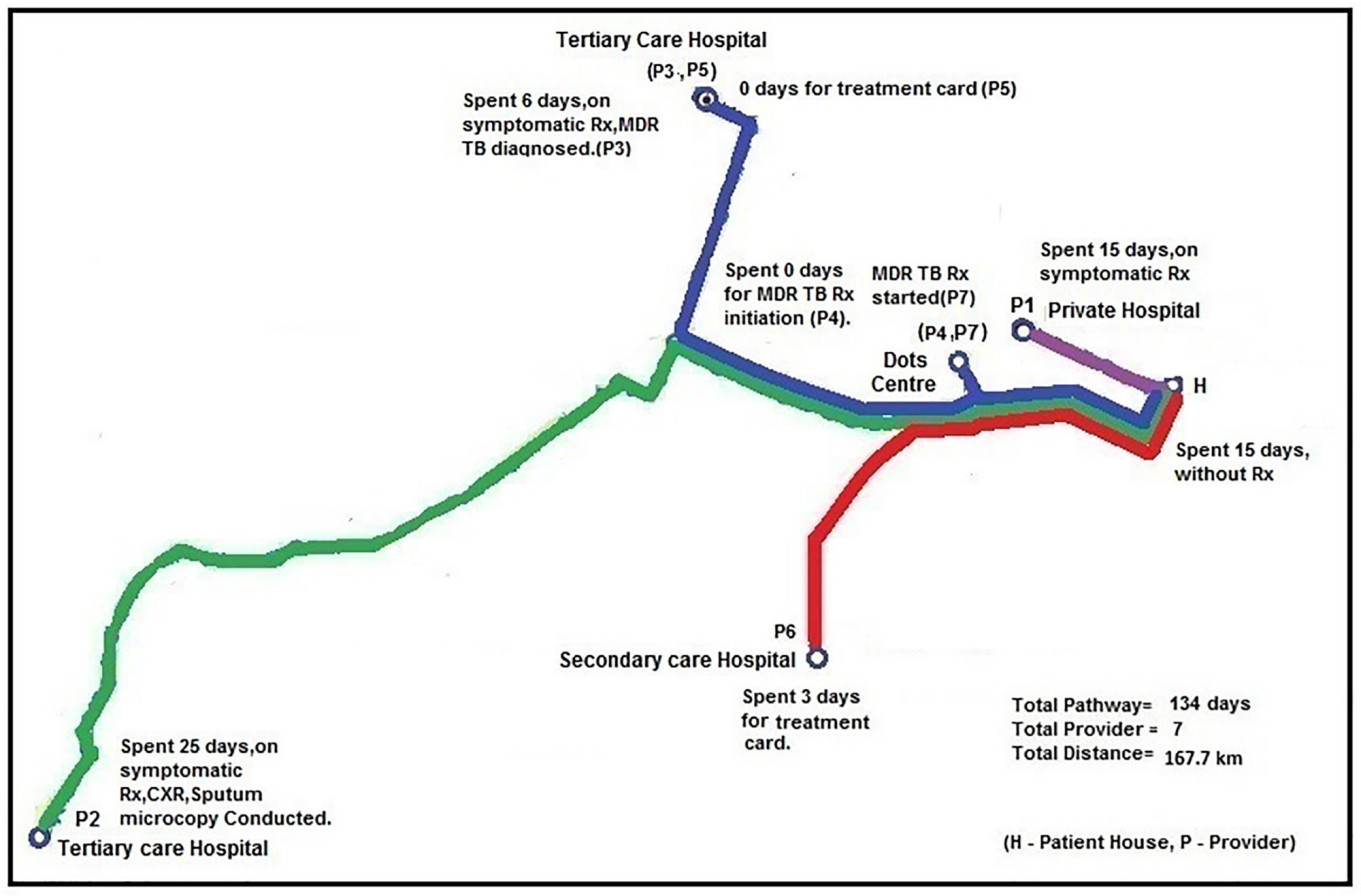
Distance travelled by Bina to access care.

However, not all patients who were referred to different facilities in the public sector were comfortable with their situation. These are quotes from some of the patients, frustrated by the referrals.

“I could not bear, so I left it (referring to a Municipal Secondary Care Hospital) on my own. …. you go early in the morning, remain standing in the queue till evening…. that too only for check-up. Medicines (are given) from another place. So when I go there, (I) spend the whole day… waste the whole day. That’s why I went back to private….”

[New Case, M, 32y, 14 months’ pathway]

“Here (at one facility) do blood test, there (at another facility) do something (some other tests), like this you run here, run there, so for this (TB treatment)… in S (Municipal Hospital) you need to run from one place to another”.

[Retreatment Case, M, 50y, 1 months’ pathway]

Vimla* *offers an explanation*, *“…here (private facility) you need to pay money for everything…*.. *So what happens*, *you pay money but things are faster*, *so no problem here*. *In municipality (-run facilities) they make you run… go here*, *go there*, *bring that form…*. *so much running around…*… *You know*, *in municipality*, *every place*, *be it R (a Municipal Secondary Care Hospital)*, *be it H (another Municipal Secondary Care Hospital)*, *… be it L (a Municipal Tertiary Care Hospital)*, *they just make you run from here to there—and because of which we get troubled”*

[New Case, F, 20y, 4.5 months’ pathway]

Patients’ pathways looked quite confusing since no two patients from the same geographical area had similar pathways, even if they had started and finished at the same facilities. And there were several reasons for such varied pathways. Often patients chose to go to a specific facility for ease of navigation and for their own convenience. However all the providers and facilities, accessed by the patients from a geographical area, did not function as part of an integrated system. This was because the PPIA had brought in only 41% of the mapped doctors and chemists in the wards it was implemented in, into its network [55] If these patients had used facilities which were part of an integrated network as recommended by McDowell and Pai in Mumbai [40], their journeys would have been less arduous. We thus echo Bloom’s [56] suggestions for health systems to identify and utilise the existing networks intelligently to make TB care pathways shorter and more efficient.

### Availability and costs of diagnostic and treatment facilities for TB

In addition to navigating the various layers of the health systems, the patient came face to face with the dilemmas of costs and payments. This was a complex issue as no two facilities operated in a similar fashion. For example, a private laboratory if approached directly for diagnostic tests, cost money but when approached through the PPIA network, these tests were done free of cost or subsidised, depending on the tests. Similarly, pharmacies which were a part of the network, provided free first line TB drugs to patients who had referral slips and vouchers from a PPIA provider. The patients were more vulnerable since there was no way for patients to have clear information on which facilities or providers to use to receive services free of cost..

Sanju* who was on treatment in the private sector said… *“A lot money was being spent*, *so I thought if this much money is going… it is a lot… 4000 rupees [USD 58*.*33] was being spent on medicines on a regular basis… I had come from the village and did not have so much money…After this someone said ‘go to* U *(a Municipal Health Post)*, *everything will be done for free’*. *…*..*(After 15 days) I went to see the doctor (the private doctor)*, *then he told me I need to do many tests*, *he wrote a whole lot of tests*, *and told me he needed all these reports*. *He said ‘There is one blood test for which the report will come after one month*, *it costs 1500 rupees [USD 21*.*87]’ Then he told me there is a system here to get all these for free—later he took time and told me everything (about the PPIA)”*.

[Retreatment Case, M, 25y, 2 months’ pathway]

Kumar’s* relative shared how expensive his treatment had been. “We did it from outside (meaning private facilities), spent 2000 rupees [USD 29.17] for tests, then the report came. They were telling it (the treatment) is very expensive—medicines and injections are very costly—each test is so expensive—some cost 9000 rupees [USD 131.24]. Then (the private doctor) said do one thing, you go to government hospital and wrote a note to (doctors at) S (Municipal TB Hospital).”

[New Case, M, 25y, 13 months’ pathway]

Several other patients had similar experiences with DR-TB treatment in the private sector, which made them stop their treatment or move to the public sector. While Firdoz* (M, 57) had been referred by the private doctor from whom he was taking his treatment, to a government facility for continuing his treatment, Simi* was not so fortunate and had to leave her treatment in between:

“(The doctor told me to) come back after one month. ….. I left him because of money. I didn’t have that much money ….. Medicines were continuing. Now (he was) asking for tests. These tests cost money… (so I thought) it is better I show it to (doctors at) H (a Municipal Secondary Care Hospital) I have (now) started (DR-TB treatment) there (at H)”.

[Retreatment Case, F, 22y, 1 months’ pathway]

### DR-TB: The ‘big-bad’ TB

Atre and Murray, [25] in their review work on DR-TB in Indian settings highlighted the role of health systems in suboptimal patient management leading to drug resistance. This is not surprising given the lack of knowledge among private practitioners regarding management of DR-TB reported by a study from Mumbai [47]. Hence, it, appears that the increasing numbers of DR-TB, while accompanied by progress in the availability of diagnostics and treatment, has not been translated into appropriate provider management practices. Consequently, poor understanding regarding DR-TB and its treatment is to be expected among patients, since health providers are one of the key sources of information for them on health matters. Our analysis offered an opportunity to understand what patients knew about DR-TB and its treatment.

Patients in our study were both aware and confused regarding MDR-TB and XDR-TB and availability of services for diagnosis and treatment in the private and public sectors. MDR-TB was seen as a ‘big’ and a dangerous version of TB and there was recognition that it needed longer treatment spanning two years, while XDR-TB was a term used in the context of requiring Bedaquiline, by a few of them.

Geetu* explains: *“MDR (TB)…*.*that is what (the doctor) said*. *MDR (TB)…*. *is (meaning needs treatment) for two years*, *that is what MDR (TB) means*. *It means it is very dangerous”*.

[New Case, F, 22y, 5 months’ pathway]

Patients, who sought help for their symptoms in the private sector, realised when they were started on treatment for their ‘big’ TB, that the treatment was expensive. Most of them could not afford to continue to pay for their medicines and shifted to the public sector. In some cases, the provider warned the patient after they were diagnosed with DR-TB, that it would be better for them to move to the public sector and receive free treatment. Majnu* (M, 32y) who had earlier shifted from a public to a private facility returned to the public sector because he could not afford private DR-TB treatment:

“This (my TB) has become big, meaning, what he (doctor) told me was it is MDR (TB). You will not be able to treat it, medicines for (treating) it are costly, so (you) should get treated at BMC (acronym for the Municipal Corporation of Mumbai).”

[New Case, M, 32y, 14 months’ pathway]

While management of DR-TB in the public sector was free, it did not appear straightforward to patients. The experiences patients narrated can be explained by the way diagnostic and treatment guidelines for DR-TB were operationalised by the public sector in Mumbai as discussed above. The inconveniences faced by the patients and the confusion in their minds were mainly because of inability on the part of providers to explain the complexities involved in diagnosis and treatment of DR-TB in a manner that patients could easily comprehend.

Nathu* said: “….. I started taking medicines there (a Municipal Health Post) for a week. Then she (the doctor) sent me to that place where all those doctors sit (referring to the Municipal TB Hospital)…. They changed my medicines and injection, because they didn’t suit me”

[Retreatment Case, M, 48y, 1 months’ pathway]

Mohan* explained: “When I got my report from J (a Private hospital), they had told me I would have to continue these medicines for two years. But … the medicines would be changed….they said ‘Among these medicines that you have been having since one month, there are two-three medicines which you are receiving as support. Now you are being offered a new medicine, for which you have to get admitted.’ I got admitted for a week after which I was started on Bedaquiline.”

[New Case, M, 19y, 3 months’ pathway]

Abu* explained how his treatment kept changing: “They (doctors) had told me (the treatment is for) six months; then they told me (treatment will be for) two years. So I started taking medicines….I must have had them (medicines) for two to three months but they didn’t suit me. While I was taking them, they checked my sputum two to three times. When they found the medicines didn’t suit me, they kept testing my sputum and then they told me to get admitted and they said they would start Bedaquiline”.

[New Case, M, 22y, 2.5 months’ pathway]

Apart from the confusion regarding DR-TB and its treatment, patients also spoke about the problems they had while on DR-TB treatment. Second line TB drugs are known to have side effects, and some patients experienced very troublesome problems. Often patients were not told what they could expect. Neither was it easy for them to get help for some of the side effects which were not dangerous, but troublesome all the same.

Abu* said: “….when I take the injection I have a reaction….in my ears. Was hearing less from one ear. And I keep scratching my ear…..see what has happened (showing his ear to the interviewer). I used to vomit….meaning I would eat the medicines and immediately vomit them out. I took the medicines for two to three months, but they were just not of any use”.

[New Case, M, 22y, 2.5 months’ pathway]

Firdoz* explained: “Because I was eating so many medicines, I had more problems… had eruptions and my head hurts”

[Retreatment Case, M, 57y, 1 months’ pathway]

Munni* complained: *“When I had a lot of reaction*, *then I told them …*..*when they started the medicines*, *they never told me anything”*.

[New Case, F, 23y, 7.5 months’ pathway]

Hasina* says: “I find it difficult to sit and get up. The injections I take, they make the nerves in my leg go tight, so I find it painful to sit”.

[New Case, F, 50y, 3 months’ pathway]

Several of these problems, were not those, for which the public health facilities offered symptomatic relief; it was only when the adverse effects were serious, did the referral mechanisms kick in to help the patient. Nevertheless the patient who was on treatment, had to live with both minor and major side effects of drugs. An empathetic health system would foresee problems and help patients get through them if they developed any due to the treatment. This would truly help them live up to Saru’s* words *‘we go to the clinic and they give medicines’;* not just give medicines, but help patients continue to live with their DR-TB.

## Conclusion

Despite having a control programme for TB, designed to follow WHO recommendations [39] from 1997, not only has the disease remained uncontrolled, but the programme in India now also has to deal with the huge challenge of managing increasing numbers of drug resistant cases [57].

A disease like TB needing long term treatment and which is managed in the private sector as well cannot be controlled merely by improving diagnosis and treatment adherence through the public health system in exclusion [25, 41, 42]. It needs a robust health system in which the public and private sectors function as equal partners supporting and complementing each other. Such a health system would be vigilant while dealing with a patient having symptoms suggestive of TB; it would have effective mechanisms in place to ensure early diagnosis, treatment initiation and completion and efficient referrals between providers and sectors; it would provide clear information to families of patients to make them understand the process, requirements and risks of treatment so that they are better prepared in their daily lives to achieve better treatment outcomes. It is only such a robust health system which can help reduce the risk of transmission of TB and development of drug resistance in the community.

All the 46 people with DR-TB included in the study were on second line treatment when they were interviewed. While they had struggled to get diagnosed and start on the correct treatment for their illness, they had finally succeeded in accessing care for their disease and were in that sense, treading a “delayed” route in the care pathway for DR-TB; their help seeking pathways are hence, pathways of so far living patients. Their struggles however provide pointers to help strengthen TB control measures and ensure they remain people- and patient-friendly.

What we see from the stories of the patients from our study is that there are different kinds of patients who straddle private and public health providers, some with previous interactions with the disease and drugs, some new patients with DR-TB, and some with XDR-TB; response to TB drugs are also not similar for all patients including side effects to drugs; lastly, there are different patterns of patient behaviour, starting from recognition of early symptoms to seeking care, all of which are influenced by sociocultural factors. These are the aspects which make implementing TB control programme a challenge, and because of which the idea of a TB free India has remained a dream.

A key aspect which this paper highlights is how each patient care pathway is different from the other and the reasons for HSB of patients are so varied. Often such patterns are simplified and are termed as ‘shopping for treatment’. However our analysis is able to delineate this pattern as ‘action-reaction’ of a patient positioned within the health systems, going round and round, seeking relief from his/her TB symptoms. These patterns need to be understood from the point of view of patients and not justified for what they simply appear to be. Patients are not ‘shopping’, rather they are going back and forth between different facilities/providers in search of a remedy. Here, at each step, the decision to ‘shop’ is based on to some extent mistrust of the provider in terms of whether the current treatment is able to create positive change (improvement in health, relief from symptoms, acceptance of diagnosis, confidence in the provider etc.). When patients perceive no improvement or are beset with unattended adverse drug effects, and they are not explained clearly why they need to take a particular drug or why they need to undergo a test, they move on to another facility/provider. Often a patient is blamed for this, but the real reason lies within the health systems. The other aspect of ‘going round and round’ is also to do with how services for TB management are laid out within the public and private health sectors [38]. In some contexts like the study setting, there are also attempts to rope in willing private providers and facilities to diagnose, treat and/or refer to public health facilities. Patients, with varying understanding of the disease and its management, gained from their own experience, their community, and health systems, and with varying capacities to bear out of pocket costs, need to travel through this complicated ‘-maze-’ of TB care.

The narratives (Sanju* and Kumar*) demonstrate the effect of high treatment costs in constraining the patient to drop out of treatment, particularly in the private sector. In 2018,a tertiary care centre in Mumbai computed costs of treatment for DR-TB patients ranging from 5700 USD to 8400 USD [58]. Additionally, these costs maybe increased in terms of extensive travel in navigating through a fragmented health system. Computing the distances travelled from symptom to treatment has yielded that patients (even those based within the limits of Mumbai) can travel upto 650km (Data not shown). This maze would be more formidable to negotiate if the patients and their families were migrants. Their pathways would be marked by uncertainties and bewilderment [59]. Therefore the make-up and the organizational aspects of any sectoral or technological initiatives need to be widely made known to communities so that they make informed choices of the provider and/or the system that they wish to access.

Communication from the patients’ perspective is another point, which the paper highlights. There have been ample IEC and BCC activities implemented by the RNTCP [60], and famous personalities in public life have been roped in to promote the programme [61]. Yet, a common person, someone from a slum in a city like Mumbai, for whom TB is not an alien condition, does not still know when to take an innocuous cough seriously, especially when cough is seen as ‘normal’ in a crowded, polluted city. Hence, even when one decides it is time to see a doctor, TB is not suspected. It is also not the first condition that providers suspect or test for, when a patient reports with cough [50]. Landing up at a doctor’s clinic, thus, does not mean the patient will have the correct road map for TB treatment. Nor does it guarantee that a provider on suspicion or diagnosis of TB will refer him/her to the appropriate facility for confirmation of diagnosis and treatment of TB, at costs which are affordable. The RNTCP, hence, needs to develop clear algorithms, drawn looking at the local health systems within a geographical area, so that irrespective of whether the patient goes to a private facility or a government facility, a general practitioner or a specialist, a new doctor in the area or one with an established practice, the patient will have a clear understanding regarding the availability of the entire gamut of screening, diagnosis, treatment and follow up services, and more importantly, s/he will be aware about the options available within the geographical area, including costs, to help exercise his/her choice in seeking TB treatment. Such an approach will not wait for the patient to falter and burden the control programme further. Further, engaging with communities and educating them about TB and the services available for diagnosis and treatment in the private and public sectors will also help ensure patients do not get conflicting health seeking advice from their community. Informed patients make better decisions, thus creating empowerment within the system and strengthening the control programme in the process.

This study is particularly important in terms of its timing when there is a heightened political willingness around End TB in India by 2025 [62], when mandatory notification for TB (even for drugs dispensed) is being instituted [9]. Recommendations from this study hence have the potential to help bring improvements in TB control not just in the city of Mumbai, or the state of Maharashtra, but in the entire nation at large. These recommendations are based on a preventative ethos which is sustainable, compared to interventions with top-down approaches, which get piloted on short term basis, but fail to show impact when scaled up.

## Supporting information

S1 File Interview guide. (PDF)
