# Supplementary material for: Such a long journey: What health seeking pathways of patients with drug resistant tuberculosis in Mumbai tell us
Source: PLoS One. 2019 Jan 17;14(1):e0209924. doi: 10.1371/journal.pone.0209924 (PMC6336307; doi:10.1371/journal.pone.0209924)
Supplement: S1 File — (PDF) [file pone.0209924.s001.pdf]

Patient Pathways to Tuberculosis Care in Mumbai – End-line study

In-depth interview guide

The Foundation for Medical Research

In collaboration with Sambodhi Research & Communications Pvt. Ltd. and Bill and Melinda Gates Foundation

24th February 2017

**INSTRUCTION: Allow the patient to narrate the replies to the following questions. Interviewers should use the probing questions as a guide for each question in order to ensure complete information on all key indicators is obtained from the respondent. Ensure that the respondent is comfortable with going forward with the interview.**
**Section A**<br>**(Patient profile)**
INTRODUCTION: Firstly, we are going to ask you about yourself and your family. By answering these questions, you will help us to understand the socio-economic issues faced by TB patients like you.<br>परिचय: सबसे पहले, हम आपके और आपके परिवार के बारे में पूछने वाले हैं। इन सवालों का जवाब देने से आप हमे आप जैसे टीबी रोगियों को पेश आ रही सामाजिक-आर्थिक मुद्दों को समझने मे मदद करेंगे।
**Questions:**
**1. Can you tell us something about yourself and your family?**<br>**१) क्या आप अपने और अपने परिवार के बारे में हमें कुछ बता सकते हैं?**
Probes: To be asked regarding the patient only (NOT Respondent)<br>1) *What is your (patient) current Age, Gender, Education, Marital status, and [Occupation before current TB episode?]**(If respondent is not the patient, also note respondent's age and gender)***<br>2) *Has your occupation changed due to the current TB episode?*<br>3) *Have you revealed your TB status at work/ school (to your employer/ at school)?*<br>4) *Family (**number of adult males and females >= 18, number of children between 6 to 18 years and number of children under 6 years' age**)*<br>5) *Number of earning members in the household?*<br>6) *Are you the primary income earner of the household?*<br>7) *What is your Religion, Mother tongue?*<br>8) *What is your knowledge of Hindi – Can you speak, write, and/or read in Hindi?*<br>9) *What is your knowledge of Marathi – Can you speak, write, and/or read in Marathi?*<br>10) *What is your Native place and how long have you been residing in Mumbai (in years)?*<br>11) *Do you consume any addictive substance/s? **If Yes**, then what?*<br>12) *Do you suffer from any chronic condition? Please specify(Multiple spontaneous response) [Probed specifically for chronic conditions - Diabetes, Hypertension, HIV/AIDS].*

13) *Have you been diagnosed with any other illness/infectious diseases such as Malaria, Dengue, Typhoid, Chikungunya etc., in the past 18 months? **If Yes**, which illness?*

14) *What colour of ration card do you possess?*

15) *Does any of your close contacts have a history of TB? **If Yes**, how many of them, and where were these contacts based – house, workplace, neighbourhood, etc?*

16) *Does any of your close contacts currently have TB? **If Yes**, how many of them, and where are these contacts based – house, workplace, neighbourhood, etc?*

दिशासुचक प्रश्न: **केवल मरीज के लिए (रिस्पॉन्डन्ट के लिए नहीं)**

१) इनके बारे में जानकारी पूछे :

रोगी की उम्र, विवाहीत/अविवाहीत, शिक्षा, लिंग और [नोकरी *ईस टी.बी के अवधी सें पहले*]?(अगर **मरीज रिस्पॉन्डन्ट** न हो तो **रिस्पॉन्डन्ट** की भी उम्र और लिंग पूछीयें।) के बारें में।

२) क्या टी.बी. के कारण आपके नौकरी पर असर पडा है? अगर हाँ, तो कैसे?

३) क्या आपने अपनी टी.बी स्थिती की जानकारी काम / स्कूल में दी है ? (आपके बॉस/प्रिंसिपल को)

४) परिवार (***number of adult males and females >= 18, number of children between 6 to 18 years and number of children under 6 years age***) ?

५)घर में आमदनी लाने वाले कितने व्यक्ती है ?

६) क्या आप घर में आमदानी लानेवाले मुख्य व्यक्ती है ?

७) आपका धर्म और मातृभाषा कौनसी है ?

८) आप हिंदी में पढ, लिख और बोल सकते है ?

९)आप मराठी में पढ, लिख और बोल सकते है ?

१०) आपका मुल निवास कौनसा है। और मुंबई में कितने सालों से रह रहे है।

११) कोई व्यसन / लत ? **(बुरी आदत-चबाने वाला तम्बाकू या धुम्रपान, शराब, किसी अन्य पदार्थ का सेवन)** अगर हाँ, तो कौनसी?

१२) पिछले १८ महीनों में क्या आपको डेंग्यु, मलेरिया, टाईफाईड, चिकनगुनिया या कोई अन्य बिमारी हुई है ? अगर हॉं, तो **कौनसी** बिमारी **थी** ?

१३) क्या रोगी को टीबी के सिवाय कोई अन्य बीमारी है जिसके लिए वह दवा खाते है ?

(एच.आय.व्ही., डायबेटीस् (शक्कर की बिमारी, मधुमेह), ब्लड प्रेशर(उच्च रक्तदाब) आदी कोई बिमारी है ?)

१४) आपके राशन कार्ड का रंग कौनसा है ?

१५) क्या आपके घर में/ नज़दीकी मे पहले **कभी किसी को** टी.बी हुआ था? अगर हाँ, तो कितने लोगो कौ? और वो कहा आधारित थे?

१६) क्या आपके घर में/ नज़दीकी मे अभी किसी को टी.बी है ? अगर हाँ, तो कितने लोगो कौ? और वो कहा आधारित है?

<u>Section B</u>

[Patient's Health Seeking Behaviour and Knowledge of TB**]**

INTRODUCTION: In the next few questions we are going to ask you about your general health care seeking behaviour and learn about your knowledge/awareness of TB. By answering these questions, you will help us to understand what you need from a health care provider and how you make choices related to health care.

प्रस्तावना: अब हम आपसे आपकी टि.बी. सम्बन्धी जानकारी के बारे में कुछ सवाल करना चाहेंगे. इन सवालों का उत्तर देकर आप ये समझने मे हमारी मदद करेंगे कि आप स्वास्थ्यकर्मियों से क्या उम्मीद करते हैं और किस प्रकार उन्हें चुनते है.

Questions:

1. **Can you please tell us in general, if you (patient) develop any illness, who is your first point of care? If it is private, then is it a private MBBS Doctor/Clinic, AYUSH practioner, Private Hospital or Nursing Home, Traditional Healer/ Quack/ Informal provider, Clinic run by NGOs, Local chemist or pharmacy? And why? If it is public, is it a Municipal/Government Hospital or a Municipal Health post? And why?**

१) अगर आप कभी बिमार हो जाएं तो आप सबसे पहले कहाँ जाते है? सरकारी या प्रायवेट और क्यो?

यदि यह प्रायवेट है, तो यह एक प्रायवेट M.B.B.S चिकित्सक / क्लिनिक, AYUSH चिकित्सक, प्रायवेट अस्पताल या नर्सिंग होम, पारंपरिक मरहम लगाने वाले / नीम हकीम / अनौपचारिक Provider है, क्लिनिक गैर सरकारी संगठनों या स्थानीय दवा की दुकान / फार्मेसी द्वारा चलाए जा रहे? और क्यों? यदि यह सरकारी है, यह एक नगर पालिका / सरकारी अस्पताल या एक नगर पालिका स्वास्थ्य पोस्ट है? और क्यों?

2. Have you heard about any TB-related initiatives in Mumbai? If Yes, then how did you hear about it?

2) क्या आपने मुंबई में कोई टीबी संबंधी programs के बारे में सुना है? यदि हाँ, तो कैसे और कहाँ सुना है?

Probe:

1) Have you heard about any initiative in the private sector that provides free medicines/diagnostic tests for TB? If **Yes,** then how did you hear about it (multiple response)? **(show pictures)**

१) प्रायवेट क्षेत्र में मुफ्त दवा/जाँच मिलती है इसके बारे में क्या आपने सुना है? यदि हाँ, तो कैसे और कहाँ? **(बहुविकल्पी) (तस्वीरें दिखाएं)**

3. Have you ever had TB in the past?

३) क्या आपको पहले भी कभी टी. बी. हुआ था ?

Probes:

1) Have you ever been diagnosed with or treated for TB in the past? If Yes, where? (Public sector, Private sector or PATH) How long were you on treatment and Did you complete the treatment?If not, why did you stop the treatment? (multiple response)? (PROBE FOR: IF PRIVATE, WAS PREVIOUS TREATMENT FREE AND NAME OF THE PROVIDER ?)

1) क्या आपने इस से पहले टीबी का जाँच/इलाज किया है? यदि हाँ, तो कहाँ? (सरकारी क्षेत्र, प्रायवेट क्षेत्र या **PATH**) कितने समय इलाज चला? और क्या आपने उपचार खतम किया ? यदि नाही तो क्यों? (अगर **पिछला** उपचार **प्रायवेट** था**, तो** क्या मुफ्त दवा मिलती थी **और डॉक्टर का नाम क्या था ?**)

**4. Can you tell us what you know about TB?**<br><br>४) क्या आप हमें बता सकते है की आप टी. बी. के बारे में क्या जानते है ?
Probes:<br><br>*1) Are you aware of any signs and symptoms of TB? **If yes**, what are 3 symptoms of TB?*<br><br>*2) How is TB transmitted (Multiple response)?*<br><br>*3) How is TB diagnosed?*<br><br>*4) Can TB be cured?*<br><br>*5) What is the length of TB treatment?*<br><br>*6) Is it possible to get TB again if you have already taken treatment once?*<br><br>*7) Are you aware of the consequences of not taking TB medicines regularly? **If Yes**, what are the consequences (Multiple response)?*<br><br>दिशासुचक प्रश्न:<br><br>१) क्या आप टी.बी. के लक्षण जानते है ? अगर हॉ, तो क्या आप टी.बी. के तीन लक्षण बता सकते है ?<br><br>२) टी.बी कैसे फैलता है ?<br><br>३) टी.बी का निदान/ जाँच कैसे किया जा सकता है ?<br><br>४) क्या टी.बी. ठीक हो सकता है?<br><br>५) टी बी के इलाज में कितना समय लगता है ?<br><br>६) क्या किसी टी बी के मरीज़ का कोर्स ख़तम होने के बाद, टी बी फिरसे हो सकता है ?<br><br>७) अगर किसी टी.बी के मरीज़ ने टी.बी की दवाईयां नियमित रूप से नहीं ली , तो उसके परिणामों के बारे में आपको पता है? यदि हाँ, तो क्या है ?
**<u>Section C</u>**

(Current TB episode - Onset of symptoms)
INTRODUCTION: In the next few questions we are going to ask you about how your illness began, and your symptoms. By answering these questions, you will help us to understand what prompted you to seek healthcare.<br>परिचय: आगे के कुछ सवालों में हम आपसे पूछना चाहेंगे कि आपके बीमारी कि शुरुवात कैसे हुई, लक्षण क्या थे. इन सवालों का जवाब देने से आप हमें यह समझने मैं मदद करेंगे की आपके स्वास्थय तलाश करने के कारण क्या थे
**Questions:**
**1. We would like to know about your illness. Can you tell us in detail about how it began and what were the symptoms that made you seek care at first?**<br>१) अभी हम आपकी बिमारी के बारे में कुछ जानना चाहते है I क्या आप बता सकते है इसकी शुरुवात कैसे हुई थी और तब क्या लक्षण दिखे थे ? कौनसे लक्षणों के कारण आप पहले स्वास्थ्यकर्मियों के पास गए?
Probes:<br>*1) What were the symptoms you developed in this current episode of TB that made you first seek care, when did it first start?* ***Date of 1st onset of symptoms****? (probe for date using a calendar)*<br>*2) Do you remember if there were any of these following symptoms – cough (> 2weeks), fever, hemoptysis, chest pain, unexplained weight loss, appetite loss, breathlessness.*<br>*3) What did you think these symptoms were due to?*<br>*4) Did you immediately go to a health care provider(doctor, chemist, etc.)?* ***If no****, why not?*<br>दिशासुचक प्रश्न :<br>**1)** वह कौनसे लक्षण थे जिसके कारण आप सबसे पहले स्वास्थ्यकर्मियों (डॉक्टर/ या दवाई की दूकान) के पास गए? इन लक्षणों कि **तारीख ? (तारीख प्राप्त करने के लिए कैलेंडर] महत्वपूर्ण दिंनो और प्रोवाइडर की प्रेस्क्रिप्शन्स का प्रयोग कीजिए)**<br>2) क्या आपको याद है कि इनमे से कोई अन्य लक्षण थे? इन के लिए पूछिए – खांसी (> 2weeks), बुखार, बलगम में खून आना, सीने में दर्द, अचानक वजन घटना , भूख की कमी, सांस लेने में तकलीफ।<br>3) आपको इन लक्षणों का कारण क्या लगा ?

4) क्या आप तुरंत किसी डॉक्टर/ या दवाई की दूकान पर गए या जाने में कोई देर की ? अगर देर कर दी तो उसकी वजह क्या थी ?
**Provider** ☐ **Section D**
**Questions on Pathway to care for the current episode of illness**<br>**(Individual provider details to be asked to the patient)**
INTRODUCTION**:** We will now talk in detail about all that you did, all the doctors you consulted and the clinics and hospitals you visited from the time you first developed symptoms till the time you were diagnosed with TB and initiated on anti-TB treatment. These questions will help us to understand why you accessed different healthcare providers for your symptoms.<br>**प्रस्तावना: अभी हम, आपमें पहला लक्षण दिखने से लेकर टी.बी. के निदान और टी.बी की दवाइयों की शुरुआत होने तक उसके चिकित्सा के लिए आपने जो भी किया, जितने प्रोवाइडरों से मिले - सलाह लि - दवाखाना और अस्पताल गए, उन सब के बारे में चर्चा करेंगे. इससे आप अपने लक्षणों के लिए क्यों अलग-अलग स्वास्थ्य-कर्मियों के पास गए इस बात को समझने में हमारी मदद होगी.**
**NOTE – Everytime Patient Changes The Provider**
Probes:<br>*1) Did you leave this provider anytime during this period? **If Yes**, then **why?***<br>*2) Did you leave the provider due to his/her consultation fee?*<br>*3) Did the provider refer you to any other provider during this time? **If Yes**, then what was the **date of leaving the provider** and **reason for the referral**?*<br>*4) Did you actually go to another/ referred provider?*<br>दिशासुचक प्रश्न :<br>१)इस दौराण क्या आपने इस प्रोवाईडर के पास जाना बंद किया ? **यदी हॉं**, तो फिर क्यों ?<br>२) क्या आपने इस प्रोवाईडर को फीस के लिए छोड़ दिया?

३) क्या इस दौराण इस प्रोवाईडर ने आपको किसी दुसरे प्रोवाईडर को रेफर किया था ? **यदी हॉं**, तो रेफरल का क्या कारण था और इस प्रोवाईडर को छोडने की **तारीख** क्या थी ?<br>४) जिनके पास आपको रेफर किया गया था /अन्यय प्रोवाईडर , क्या आप उनके पास गये थें ?
**<u>Section D</u>**<br>**(General Provider Information and Symptomatic Treatment)**
**Questions:**
**1. When you decided to get some treatment for your symptoms, whom did you consult first? Can you give us some details about this provider and facility?**<br>**१) जब आपने अपने लक्षणों का उपचार करने कि सोची तब उसके लिए आप पहले किस स्वास्थ्य-कर्मियों के पास गए? क्या आप उस स्वास्थ्य-कर्मियों या उस सुविधा के बारेमें कुछ बता सकते हैं ?**
Probes:<br>*1) Can you tell us the Name, Qualification, Name of the facility, Location of the clinic/ hospital/chemist?*<br>***2) When did you first approach this provider/What was the date of approaching this provider? (Use Calendars or significant events or prescription to obtain date)***<br>*3) Can you tell us about the reason/s why you approached this provider?*<br>*4) Did the provider ask about any current/past history of TB among your family members/ close contacts?*<br>*5) Did you inform the provider about any current/past history of TB among your family members/ close contacts?*<br>*6) Did the provider ask you about your past history of TB?*<br>*7) Did you inform the provider about your past history of TB? (**Ask ONLY for retreatment cases)***<br><br>दिशासुचक प्रश्न :<br>१) उस प्रोवाईडर का नाम, डिग्री, पता क्या था ? क्या प्रोवाइडर औपचारीक, उनौपचारीक या कैमिस्ट था ? और वह किस तरह की सुविधा थी ? और सुविधा का नाम?

२) इनसे पहली बार मिलने की तारीख बता सकते हैं ? **(तारीख प्राप्त करने के लिए कैलेंडर, महत्वपूर्ण दिंनो और प्रोवाइडर की प्रेस्क्रिप्शन्स का प्रयोग कीजिये)**<br>३) इस प्रोवाईडर के पास जाने का क्या कारण था ?<br>४) क्या प्रोवाईडर ने आपको पुछाँ था की आपके परिवार /नज़दीकी में पहले भी कभी किसी को टी.बी हुआ था या इस समय भी किसी को टीबी है ?<br>5) क्या आपने प्रोवाईडर को बताया था की आपके परिवार /नज़दीकी में पहले भी कभी किसी को टी.बी हुआ था या इस समय भी किसी को टीबी है ?<br>६) क्या प्रोवाईडर ने आपका पुछाँ था की आपको पहले भी टी. बी हुआ था ?<br>७) क्या आपने प्रोवाईडर को बताया था के आपको पहले भी टी. बी हुआ था ?**(Ask ONLY for retreatment cases)**
**2. Can you now tell us about any medication/ treatment given by this provider/facility (use the name the respondent uses) prior to/simultaneously/after to being asked to undergo the tests that were advised?**<br>**२) आपको कोई भी टेस्ट लिखने से पहले/ के समय / के बाद क्या उस प्रोवायडर ने आपको कोई दवा या दवाइयां लिखी थी ?**
Probes:<br>*1) Did the provider advise any tests?*<br>*2) Did the provider give you any symptomatic medication/treatment?If yes was it* ***before, simultaneously*** *or* ***after*** *ordering the tests?*<br>दिशासुचक प्रश्न :<br>१) आपको प्रोवाईडरने कोई भी टेस्ट करने को कहा था?<br>२) आपको प्रोवाईडर ने लक्षण कम करने के लिए कोई भी दवाइयां दलिखी थी ? टेस्ट/ जाँच लिखने से पहले, बाद में या एक साथ?
**<u>Section E</u>**<br>**(Test/s ordered by the provider/s you visited**)

Questions:

1. **Can you tell us if a) chest x-ray, b) GeneXpert, c) sputum microscopy or d) Any other test/s were recommended by this provider/facility (use the name the respondent uses)? Kindly elaborate on each of the tests advised and facilities where they were conducted.**

१)क्या इस प्रोवाईडर ने आपको **A)** छाती का एक्स-रे **B)**जीन एक्सपर्ट **C)** बलगम माइक्रोस्कोपी या **D)** कोई और टेस्ट लिख कर दिया था ? (रोगी जो नाम लेता है उसी नाम का इस्तमाल करें) क्या आप हमें इन टेस्टस् के बारे में और जानकारी दे सकते हैं।

Probes:

1) *Did the provider advise you to undergo any tests such as a) chest x-ray, b) GeneXpert, c) sputum microscopy, d) Other tests etc.?*

दिशासुचक प्रश्न :

१) क्या इस प्रोवाईर ने आपको A) छाती का एक्स-रे B) जीन एक्सपर्ट C) बलगम माइक्रोस्कोपीया D) कोई और टेस्ट लिख कर दिया था ?

If Chest X-ray was advised then:

1) When was the CXR advised? Date of advising? ***(Try to get the exact date using calendars, lab reports, etc.)***

2) Were you given any referral slip/voucher(coupon or message) by the provider for the chest x-ray?

3) Did you get the x-ray done as advised? ***If No****, then why not?*

4) ***If Yes****, then what was the* ***date of conducting test****?* ***(Check documents – lab reports, etc. or use calendar)***

5) Did you utilise the referral slip/voucher for getting the CXR done?

6) Where did you get it done (try to get the exact name of the laboratory)? What was the reason for getting it done at this laboratory (mention name of the lab/facility used by the patient)?

7) Did you pay for the x-ray?

8) On what date did you collect the chest x-ray report?

9) Were you diagnosed with TB based on your CXR findings?

दिशासुचक प्रश्न :

1) CXR की सलाह कब दी थी ? **तारीख** क्या थी ?**(तारीख प्राप्त करने के लिए कैलेंडर, महत्वपूर्ण दिंनो और प्रोवाइडर की प्रेस्क्रिप्शन्स का प्रयोग कीजिये)**

२) क्या प्रोवाइडर ने छाती के एक्सरे- के लिए आपको कोई वाउचर /रेफरल स्लिप *(coupon or message)* दी थी ?

३) क्या आपने वह एक्स-रे करवाया ? **यदी नही**, तो क्यों नही ?

४) **यदी हॉं**, तो टेस्टे करवाने के लिए बताये जाने पर आपने कितने दिनो बाद वह टेस्ट करवाया ? CXR करवाने की तारीख क्या थी? **(दस्तावेजों की जाँच करें - प्रयोगशाला/ लैब की रिपोर्ट, आदि या कैलेंडर का उपयोग करें)**

५) क्या आपने एक्स-रे करवाने के लिए रेफरल स्लिप / वाउचर का उपयोग किया था?

६) कहॉं से करवाया ?वहां करवाने का कारण क्या था ? (प्रयोंगशाला का सही नाम जानना जरुरी है)

७) क्या आपने एक्स-रे के पैसे भरे थे ?

८) आपने एक्स-रे की रिपोर्ट किस तारीख को इकठा की थी ?

९) क्या यह एक्स - रे के रिपोर्ट को देखने के बाद ,डॉक्टर ने आपको बताया की आपको टी.बी हुआ है ?

If GeneXpert (GX) was advised then:

1)When was the GX advised? Date of advising? ***(try to get the exact date using calendars, lab reports, etc.)***

2) Were you given any referral slip/voucher(coupon or message) by the provider for the GX?

3) Was GX conducted as advised? ***If No****, then why not?*

4) ***If Yes****, then what was the* ***date of conducting test? (Check documents – lab reports, etc. or use calendar)***

5) Did you utilise the referral slip/voucher for getting the GX done?

6) Where did you get it done (try to get the exact name of the laboratory)? What was the reason for getting it done at this laboratory (mention name of the lab/facility used by the patient)?How many samples were collected?

7) Was your sputum sample collected and transported by a field officer/Did you have to drop/leave your sputum sample at the provider's office or at the laboratory?

8) Did you pay for the GX?

9) On what date did you collect the report?

10) Were you diagnosed with TB based on your GX findings?<br><br>दिशासुचक प्रश्न :<br>1) जीनएक्सपर्ट की सलाह कब दी थी ? **तारीख** क्या थी ?**(तारीख प्राप्त करने के लिए कैलेंडर, महत्वपूर्ण दिंनो और प्रोवाइडर की प्रेस्क्रिप्शन्स का प्रयोग कीजिये)**<br>२) क्या प्रोवाइडर ने जीनएक्सपर्ट के लिए आपको कोई वाउचर / रेफरल स्लिप*(coupon or message)* दी थी ?<br>३) क्या आपने वह जीनएक्सपर्ट करवाया ? **यदी नही**, तो क्यों नही ?<br>४) **यदी हाँ**, तो टेस्टे करवाने के लिए बताये जाने पर आपने कितने दिनो बाद वह टेस्ट करवाया ? जीनएक्सपर्ट करवाने की तारीख क्या थी?**(दस्तावेजों की जाँच करें - प्रयोगशाला/ लैब की रिपोर्ट, आदि या कैलेंडर का उपयोग करें)**<br>५) क्या आपने जीनएक्सपर्ट करवाने के लिए रेफरल स्लिप / वाउचर का उपयोग किया था?<br>६) कहॉ से करवाया ?वहां करवाने का कारण क्या था ? (प्र्योंगशाला का सही नाम जानना जरुरी है)<br>७) क्या आपका बलगम कोई जाँच के लिये लेके गया था? बलगम कितनी बार लिया गया था?<br>८) क्या आपने जीनएक्सपर्ट के पैसे भरे थे ?<br>९) आपने जीनएक्सपर्ट की रिपोर्ट किस तारीख को इकठा की थी ?<br>१०) क्या यह जीनएक्सपट के रिपोर्ट को देखने के बाद ,डॉक्टर ने आपको बताया की आपको टी.बी हुआ है ?
<u>If Sputum Microscopy was advised then:</u><br>*1) When was the sputum microscopy advised? Date of advising?* ***(try to get the exact date using calendars, lab reports, etc.)***<br>*2) Was sputum microscopy conducted as advised?* ***If No****, then why not?*<br>*3)* ***If Yes****, then what was the* ***date of conducting test (Check documents – lab reports, etc. or use calendar)****?*<br>*4) Where did you get it done (try to get the exact name of the laboratory)? What was the reason for getting it done at this laboratory (mention name of the lab/facility used by the patient)?*<br>*5) On what date did you collect the report?*<br>*6) Were you diagnosed with TB based on your sputum microscopy findings?*

दिशासुचक प्रश्न :<br>1) बलगम माइक्रोस्कोपी की सलाह कब दी थी ? **तारीख** क्या थी ?(**तारीख प्राप्त करने के लिए कैलेंडर, महत्वपूर्ण दिंनो और प्रोवाइडर की प्रेस्क्रिप्शन्स का प्रयोग कीजिये)**<br>२) क्या आपने वह बलगम माइक्रोस्कोपी करवाया ? यदी नही, तो क्यों नही ?<br>३) **यदी हॉं**, तो टेस्टे करवाने के लिए बताये जाने पर आपने कितने दिनो बाद वह टेस्ट करवाया ? बलगम माईक्रोस्कोपी करवाने की तारीख क्या थी? **(दस्तावेजों की जाँच करें - प्रयोगशाला/ लैब की रिपोर्ट, आदि या कैलेंडर का उपयोग करें)**<br>४) कहॉं से करवाया ?वहां करवाने का कारण क्या था ? **(प्रयोंगशाला का सही नाम जानना जरुरी है)**<br>५) आपने बलगम माइक्रोस्कोपी की रिपोर्ट किस तारीख को इकठा की थी ?<br>६) क्या यह बलगम माइक्रोस्कोपी के रिपोर्ट को देखने के बाद ,डॉक्टर ने आपको बताया की आपको टी.बी हुआ है ?
If any other test was advised then:<br>*1) Name of the test/s?*<br>*2) What happened after that?*<br>दिशासुचक प्रश्न :<br>१) टेस्ट का नाम ?<br>२)उसके बाद क्या हुआ?
**Section F**<br>**(TB Diagnosis)**
**1. Let us now talk about what happened after you went to this provider with your test results. What did he say you were suffering from after seeing the reports and did you continue with this provider after that?**

१) अब जब आप अपने टेस्ट के रिपोर्ट इस प्रोवाईडर के पास लेकर गए तब क्या हूआ , हम उनके बारें में बात करेंगे। टेस्ट के रिपोर्ट देखने के बाद इस प्रोवाईडर ने क्या कहाँ कि आपको किस चीज की पीडा है ? इसके बाद भी क्या आप इसी प्रोवाईडर के पास ही जा रहे थे ?
Probes:<br>1*) When did you approach the provider with your test results?* **(try to get the exact date based on calendar, lab reports, etc.,)**<br>*2) Did the provider make any diagnosis?**If Yes**, then what was diagnosed?*<br>*3) Did the provider make a diagnosis of TB? **If Yes**, what was the **date of TB diagnosis**? **(Use Calendars or significant events or test reports to obtain date)***<br>*4) What happened after that?*<br><br>दिशासुचक प्रश्न :<br>१) फिर आपने प्रोवाईडर को रिपोर्ट कब दिखाई? इसकी तारीख क्या थी ? (**तारीख प्राप्त करने के लिए कैलेंडर, महत्वपूर्ण दिंनो और प्रोवाइडर की प्रेस्क्रिप्शन्स का प्रयोग कीजिये)**<br>२) क्या प्रोवाईडर ने कोई निदान किया? **यदी हॉं**, तो फीर क्या ?<br>३) क्या प्रोवाईडर ने टी.बी का निदान किया था ? **यदी हॉं**, तो फिर इसकी तारीख क्या थी ? (**रोगी के दस्तावेज, प्रयोगशाला के रिपोर्ट या कैलेंडर का इस्तमाल कीजीएँ**)<br>४)उसके बाद क्या हुआ?
**<u>Section G</u>**<br>**(TB Treatment)**
**1. Can you tell us about the medical treatment you received after you were diagnosed with (use name of the condition the respondent/patient was told to be suffering from) by this provider/facility? (use the name respondent uses)**<br>१) टी.बी का निदान करने बाद क्या आपको इस प्रोवाईडर/सुविधा द्वारा मेडीकल इलाज मिला था ? क्या आप हमें इसके बारे में बता सकते हैं?

Probes:

*1) Was treatment for TB started after the diagnosis? **If Yes**, then what was the **date of treatment initiation? (Use Calendars or significant events or treatment card to obtain date)***

2) How long was your entire treatment course advised?

3) What drugs were given (note down all drugs mentioned by patient)?

*4) Was any referral slip/voucher(message or coupon) provided for the TB drugs? **If yes**, then did you utilise the referral slip/voucher for getting the TB drugs?*

5) Did you pay for the TB drugs?

*6) Did you complete your entire TB treatment?**(Yes/No/Ongoing)***

*7) If treatment was **not completed**, why didn't you complete the treatment?*

*8) Did the provider ask you to repeat any test?**If YES**, then which(Multiple response)*

*9) Did you have any follow-up visits? **If Yes**, then what were the fees for the follow up visits?*

*10) Were any follow up CXR advised? **If Yes,** then did you pay for the follow up CXR?*

*11) Were any follow up sputum microscopy advised? **If Yes,** then did you pay for the follow up sputum microscopy?*

12) If the treatment was incomplete, did you continue your treatment till you approached the next provider? **(Only if the treatment is incomplete)**

दिशासुचक प्रश्न :

१) टी.बी का निदान करने के बाद क्या प्रोवाइडर द्वारा उसका इलाज शुरु किया गया? **यदी हॉं**, तो फिर इलाज किस तारीख को शुरु किया गया ? **(तारीख ढुडने के लिए कैलेंडर और ईलाज कार्ड का इस्तमाल कीजीए)**

२) रोग के इलाज की अवधि का समय क्या बताया गया था?

३) कौनसी दवाईयॉं दी गई थी (सभी टी.बी की दवाईयों के और अन्य दवाईयों के नाम पूछे)

४) टी.बी की दवाईयों के लिए क्या आपको कोई रेफरल स्लिप / वाउचर*(message or coupon)* दी गई थी ?**यदी हॉं,** तो फिर क्या आपने रेफरल स्लिप / वाउचर का उपयोग किया था?

५) क्या आपने टी. बी दवाईयों के लिए पैसे भरे थे ?

६) क्या आपने टी.बी का इलाज पुरा किया था ? (**हॉं/ना/अभी भी चल रहा है**)

७) यदी आपने इलाज पुरा नही किया तो इसका क्या कारण था ?

८) क्या प्रोवाईडर ने आपको कोई टेस्ट दोहराने के लिए कहा? **यदी हॉं**, तो फिर कौनसा ?<br><br>९) क्या आप उपचार के दौरान प्रोवाइडर से मिलते रेहते थे ?**यदी हॉं**, तो फिर आपने कितने पैसे भरे थे?<br><br>१०) क्या आपका इस दौरान छाती का एक्स - रे होता रहा? **यदी हॉं**, तो फिर क्या आपने पैसे भरे थे?<br><br>११) क्या आपका इस दौरान बलगम का जाँच / माइक्रोस्कोपी हुआ ? **यदी हॉं**, तो फिर क्या आपने पैसे भरे थे?<br>१२) जब आपने ईस प्रोवाईडर को छोड़ दिया तब क्या आपने अपनी दवाई जारी रखी? **(Only if the treatment is incomplete)**
**<u>Section H</u>**<br><br>**(MDR-TB Diagnosis and Treatment)**
**1. Can you tell us if the provider changed your medications? If yes, then what was the reason?**<br><br>१) क्या प्रोवाईडर ने आपकी दवाईयॉं बदली थी ? यदी हॉं, तो इसका कारण क्या था ?
Probes:<br>*1) Was MDR-TB diagnosed? **If Yes**, what was the **date of diagnosis? (Use Calendars or significant events or test reports to obtain date)** - Did the provider change your treatment? Did he inform you about the reason for change?*<br>*2) Which test was used to detect MDR-TB? (DST, Sputum culture, LPA etc.)*<br>*3) Were any PTE tests advised? (pack of 11 tests)*<br>*4) Was MDR-TB treatment given? **If Yes**, what was the **date of treatment initiation? (Use Calendars or significant events or treatment card to obtain date)***<br>*5) How long was/is your entire MDR-TB treatment course advised for (in days)?*<br>*6) What drugs were given (note down all drugs mentioned by patient – TB and other drugs)?*<br><br>दिशासुचक प्रश्न :<br>१) क्या MDR-TB का निदान हुआ था ? **यदी हॉं**, तो फिर निदान की तारीख क्या थी ? (**कैलेंडर तारीख ढुंडने के लिए और इलाज कार्ड का इस्तमाल किजीए**) क्या प्रोवाईडर ने आपकी दवाईयॉं बदली थी ? क्या प्रोवाईडर ने दवाईयॉं बदलने का कारण आपको बताया था ?<br>२) कोई टेस्ट किया गया था ? (जैसे की *DST*, *Sputum culture, LPA* etc.)

३) निदान होने के बाद और ईलाज के पहले क्या और भी टेस्ट हूए थे?

४) क्या MDR-TB का इलाज दिया गया थी ? **यदी हॉ**, तो फिर इलाज शुरु करने की **तारीख** क्या थी ? (**तारीख ढुंढने के लिए कैलेंडर और इलाज कार्ड का इस्तमाल किजीए**)

५) आपकी MDR-TB के पूर्ण इलाज की अवधि क्या थी /हैं ?(दिनों में)

६) कौनसी दवाईयॉ दी गई थी ? (सभी टी.बी की दवाईयों का और अन्य दवाईयों के नाम पूछे)

Section I

(Advice and Counselling)

TO BE FILLED FOR DIAGNOSING AND TREATING PROVIDER OF TB AND MDR TB

1. **Can you tell us about the advice and counselling related to TB provided by this provider / compunder/ field officer (use the name respondent uses) prior to start of the treatment/during the treatment course? (For each provider who diagnosed and/or treated TB and MDR-TB)**

१) टी.बी का इलाज शुरु होने से पहले / इलाज के दोरान आपको इस प्रोवाईडर / कंपाउंडर / field officer ने क्या सलाह और काऊॅंसिलिंग दी थी, क्या आप हमें उसकी जानकारी दे सकते है ? (यह प्रष्ण प्रत्येक प्रोवाईडर के लिए उपयुक्त है जिसने टी बी और MDR-TB का निदान और/या इलाज किया)

Probes:

*1) Did the provider advice you to get tested for any other illnesses/ conditions like HIV/ Diabetes? **If yes, then which one?***

2) What kind of information was given to you/your family by the provider/ compounder/ field officer on the following?

a) importance of adherence

b) adverse drug reactions

c) dietary advice

d) precautions to avoid transmission – cough etiquette

*3) Did the provider/ compounder/ field officer give you any specific advice regarding (if any) children under 6 in your household? **If Yes**, then what was it?*

*4) Did the provider advise a chest x-ray as a follow-up test? **If Yes**, then how often was it advised?*

*5) Did the provider advise a sputum microscopy as a follow-up test? **If Yes**, then how often was it advised?*

दिशासुचक प्रश्न :

१) क्या प्रोवाईउर ने आपको एच आय व्ही / मधुमेह (सुगर की बिमारी) आदि बिमारीयो की जॉच करवाने की सलाह दी थी ?**यदी हॉ**, तो कौन कौन से?

२) प्रोवाईउर / कंपाउंडर/ Field officer ने आपको इन विषयो पर क्या सलाह दी ?

अ) टी.बी की नियमित रूप से दवाईयां नहीं लेने पर क्या हो सकता है ?

ब) दवाईयों की दुष्प्रभाव / हानिकरक प्रभाव

सी) आहार से संबंधित सलाह

डी) टी.बी का प्रसार नही हो इसलिए- खासी शिष्टाचार / तहज़ीब

३) क्या प्रोवाईडर / कंपाउंडर/ Field officer ने आपको ६ साल से नीचे के बच्चों के साथ केसे रेहना चाहिए/ सम्बंधितं कोई सलाह दी ? **यदी हॉ**, तो क्या सलाह दी ?

४)क्या प्रोवाईडर ने आपको फोलो-अप के लिए छाती का एक्स-रे निकालने की सलाह दी थी ?**यदी हॉ**, तो किनी बार बोला ?

५)क्या प्रोवाईडर ने आपको फोलो-अप के लिए बलगम माइक्रोस्कोपी करवाने की सलाह दी थी ?**यदी हॉ**, तो किनी बार बोला ?
